# Archeological data with AI- and physics-based modeling explain typhoon-induced disasters in inland China around 3000 yr B.P.

**DOI:** 10.1126/sciadv.aeb1598

**Published:** 2026-03-04

**Authors:** Ke Ding, Siyang Li, Aijun Ding, Houyuan Lu, Jianping Zhang, Dazhi Xi, Xin Huang, Sijia Lou, Xiaodong Tang, Xin Qiu, Lejun He, Yue Ma, Haoxian Lin, Shiyan Zhang, Derong Zhou, Xiaolu Zhou, Zhe-Min Tan, Congbin Fu, Quansheng Ge

**Affiliations:** ^1^ State Key Laboratory of Severe Weather Meteorological Science and Technology, School of Atmospheric Sciences, Nanjing University, Nanjing 210023, China.; ^2^ Institute of Geographic Sciences and Natural Resources Research, Chinese Academy of Sciences, Beijing 100101, China.; ^3^ School of History, Nanjing University, Nanjing 210023, China.; ^4^ Jiangsu Provincial Innovation Center for Climate Change, Nanjing 210023, China.; ^5^ Frontiers Science Center for Critical Earth Material Cycling, Nanjing University, Nanjing 210023, China.; ^6^ State Key Laboratory of Lithospheric and Environmental Coevolution, Institute of Geology and Geophysics, Chinese Academy of Sciences, Beijing 100029, China.; ^7^ Department of Earth Science, The University of Hong Kong, Hong Kong 999077, China.

## Abstract

Climate change–related extreme events during the mid-late Holocene, especially around 3000 years before the present (yr B.P.), severely threatened human survival and cultural development at various locations. However, although marked social change during this period in China have also been reported to coincide with extreme disasters, the causes and impacts of these events remain unclear. Here, we aligned paleoclimate reconstructions with quantitative analyses of archeological evidence, including oracle bone scripts, together with artificial intelligence– and physics-based model simulations to uncover the causes. We found that intensified typhoon activities exerted considerable impacts on climate extremes and social change in inland China around 3000 yr B.P. These findings underscore the urgent need to improve preparedness for today’s typhoon-induced disasters in the context of accelerating climate change.

## Teaser

Intensified typhoons caused extreme rainfall and floods in inland China, threatening cultural development, around 3000 yr B.P.

## INTRODUCTION

Extreme weather events in the context of climate change have raised increasing concerns in recent years for causing severe disasters worldwide (*1*). Similarly, during the short period of marked climatic transition in the mid-late Holocene, a series of extraordinary events, such as that related to the severe El Niño–like conditions around 3300 years before the present (yr B.P.), took place at various regions (*2*–*4*). Some of these events have been reported to pose substantial threats to people and their cultures, potentially contributing to social disruptions, crises, and even the decline of ancient civilizations (*2*, *5*–*6*). Almost in this same period, civilizations in China, such as the Shang Dynasty (3550 to 2996 yr B.P., centered in the Central Plains of China), Sanxingdui, and Shi’erqiao cultures (~3700 to 3000 and 3000 to 2500 yr B.P. in the Chengdu Plain), also suffered from severe climate events and abrupt social changes in their flourishing times (*7*–*10*). However, although a rapid mid-late Holocene climate change in China has been reported in varied paleoclimatic reconstructions (*11*–*13*), climate extremes, along with their causes, dynamics, and consequences on human civilizations, remained difficult to establish. These lines of inquiry have been partly hobbled by the lack of well-documented climatic and meteorological conditions during this period and the resulting lack of understanding of the meteorological process of these extreme events.

In this study, we quantitatively examined the weather-related written records in ~55,000 pieces of oracle bone scripts (3200 to 2996 yr B.P.), the earliest form of systematic Chinese writing (*14*–*15*), and developed several indexes representing the climate conditions during ~200 years in the Late Shang Dynasty. Meanwhile, a more detailed reconstruction of social development during the Chinese Bronze Age was inferred mainly from changes in the relative population size in the Central Plains of China (referred to below as the Central Plains, known as the “cradle of Chinese civilization”) and from variations in the number of archeological sites in the Chengdu Plain, where the Sanxingdui culture of the ancient Shu civilization was located. On this basis, we aligned our reconstructed indexes of climate variations and our identified population and social changes with archeological and documentary evidence of climate extremes and with existing high-resolution regional paleoclimate records. What stood out here was intensified typhoon activities, which presented as a potentially influential factor in the climate extremes, as well as the population and social changes in the Central Plains and the Chengdu Plain during this period. To further assess the impact of intensified typhoon activities on the extreme events, we combined modern meteorological analysis and model simulations, especially the artificial intelligence (AI)–based model “Pangu-Weather” (*16*) in our study. Together, these data and analyses allow an interdisciplinary exploration of how intensified typhoon activities exerted unexpected disastrous influences in inland China during the Bronze Age. These findings underscore the importance of considering potential threats of typhoon-related climate extremes when understanding the interplay between climatic conditions and social changes, even in inland areas.

## RESULTS

### Reconstruction of population and social changes

**Fig. 1.**
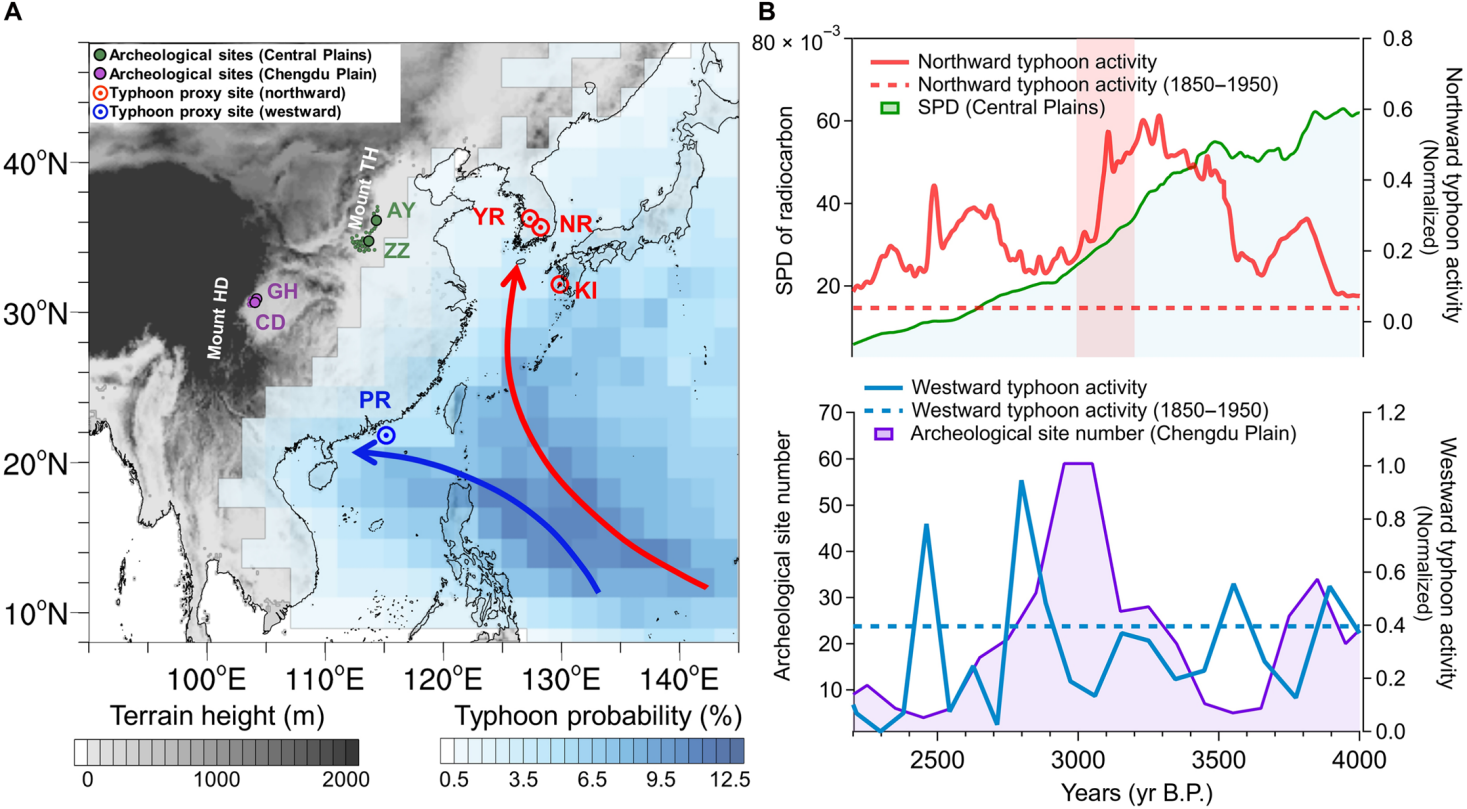
Temporal changes in typhoon activity, population size (Central Plains), and archeological site number (Chengdu Plain) during the mid-late Holocene. (**A**) Distribution of typhoon probability in modern times (1950 to 2021) and the locations of archeological sites and paleo-typhoon proxies used in this study. YR, NR, KI, and PR represent the Yugu River (*24*), the Nakdong River (*25*) in South Korea, the Kamikoshiki Island (*23*) in southwestern Japan, and the Pearl River Estuary (*26*) in South China, respectively. The thick red (blue) solid arrow line denotes the prevailing northward (westward) typhoon tracks. (**B**) Fluctuations in normalized northward typhoon activities and variations in population size in the Central Plains [represented by the reconstructed summed probability distributions (SPD); top]; fluctuations in westward typhoon activities (*26*) and the temporal distribution of archeological sites in the Chengdu Plain (bottom). The northward (westward) typhoon activities were normalized from the typhoon proxies got in YR, NR, and KI (PR). The red (blue) dashed lines give the northward (westward) average typhoon activities during 1850 to 1950. The time range of oracle bone scripts (*14*) is indicated by the red translucent strip on the top panel.

Civilizations in the Central Plains and Chengdu Plain created splendid and thriving bronze age cultures during the mid-late Holocene in China (*17*–*18*). As shown in Fig. 1 and fig. S1, archeological sites of mid-late Holocene civilizations in the Central Plains were located in a flat terrain to the east of mountains [including Mount Taihang (TH)], with Zhengzhou (ZZ) and Anyang (AY) as capital cities of the Shang Dynasty (*15*). Meanwhile, archeological sites in the Chengdu Plain, such as Sanxingdui site in Guanghan (GH) and Jinsha site in Chengdu (CD), were also located to the east of mountains, which belong to the Hengduan (HD) Mountains. Therefore, civilizations in these two areas, owing to the geographical features, tend to benefit from abundant orographic rainfall, but, at the same time, being sensitive to extreme rainfall events and the induced flood disasters (*19*–*21*).

On the basis of the datasets of radiocarbon dates in China (*22*), we constructed the summed probability distributions (SPD) of ^14^C data for the archeological sites in the Central Plains during the mid-late Holocene, which serves as a proxy of regional population dynamics here (see Materials and Methods). For the Chengdu Plain, an examination of episodical variation in number of archeological sites (table S1 and fig. S2) in this study helps indicate population dynamics, or even social peaks and downturns in this region, due to the lack of sufficient radiocarbon dates here. As can be seen in Fig. 1B, the population-related indicators in the Central Plains and in the Chengdu Plain both experienced several abrupt downturns during the period between 4000 and 2500 yr B.P. in the mid-late Holocene, along with certain differences in the specific time points. Notably, the population fluctuations in the Central Plains and in the Chengdu Plain coincided very well with the reconstructed variations in northward and westward typhoon activities (*23*–*26*), respectively (Fig. 1B and figs. S3 and S4).

When northward typhoon activities notably intensified around 3800 and 3300 yr B.P., archeological cultures in the Central Plains witnessed rapid declines in their population sizes. While in the Chengdu Plain, a sharp reduction in the number of archeological sites coincided perfectly with the abrupt intensification of westward typhoon activities around 2800 yr B.P., along with smaller-scale downturns here, showing good correlations with intensified westward typhoon activities around 3600 and 2450 yr B.P. In the meantime, archeological and historical records also suggest notable social changes happened in the Central Plains and Chengdu Plain during periods of intensified typhoon activities, such as transitional phases between major archeological cultures, frequent capital relocations, and decrease in regional levels of settlement hierarchy (Supplementary Text and fig. S5) (*7*, *14*, *27*–*32*). Given the disastrous impact of typhoon and its correspondent meteorological conditions in inland China presented in modern cases for inducing extreme rainfall and floods (table S2) (*33*–*35*), we aimed to determine whether typhoons were a key factor in causing climate extremes and impeding social development during the mid-late Holocene.

### Reconstruction of typhoon-related inland climate extremes

#### Correlation between northward typhoon and extreme rainfall in the Central Plains

**Fig. 2.**
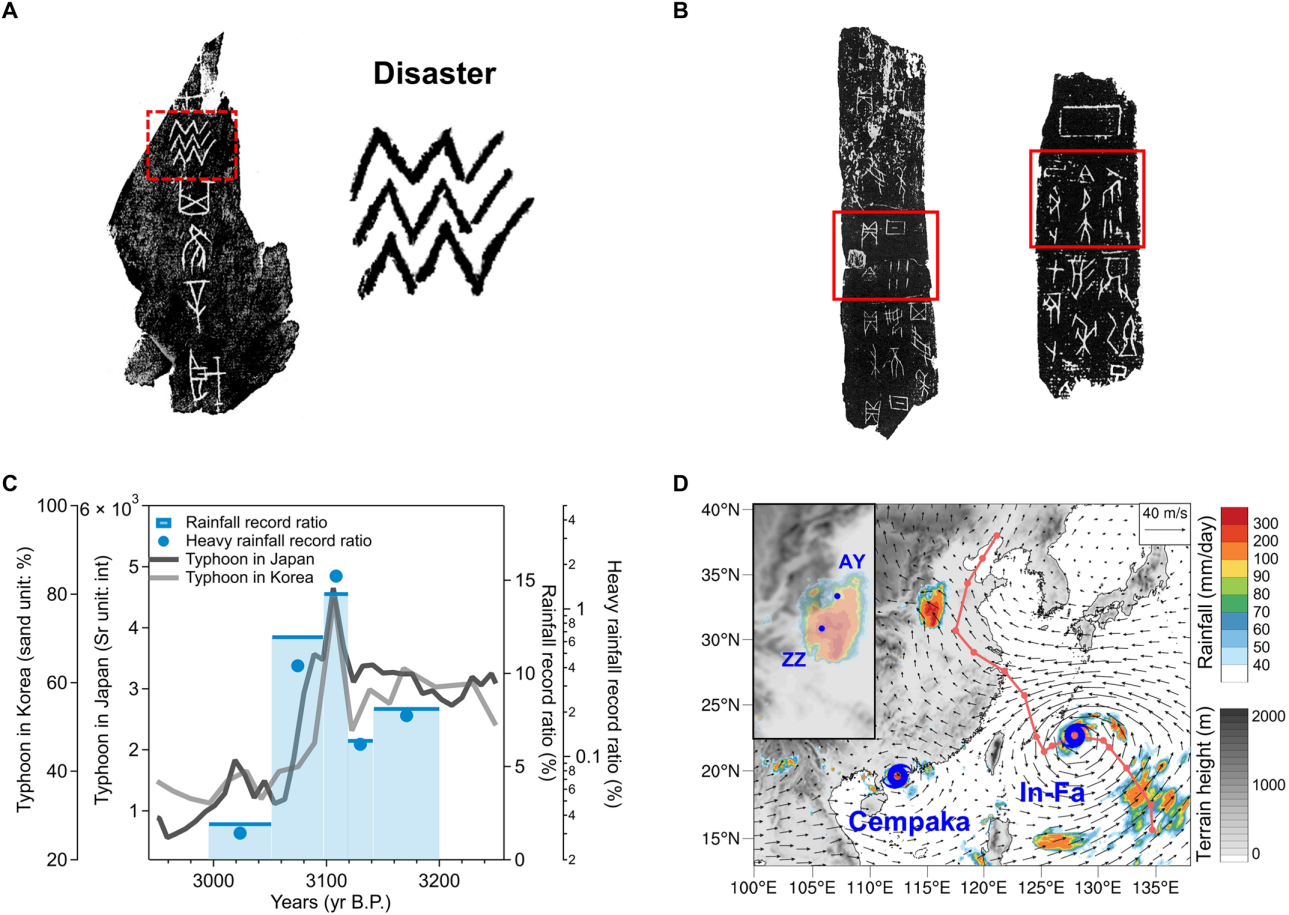
Floods and rainfall situations recorded in oracle bone scripts in the Central Plains and their correlation with northward typhoon activities. (**A**) Rubbing of oracle bone script piece (He 12836; front side); image reproduced from M. Guo (*92*), used with permission of Zhonghua Book Company. Translation: “Will there be a disaster?…….” The character representing disaster, highlighted by the red dashed box, is shown beside the rubbing. (**B**) Rubbings of typical oracle bone script pieces that contain (upcoming) rain (left; He 9757) and (upcoming) heavy rain (right; He 27219); images reproduced from M. Guo (*92*), used with permission of Zhonghua Book Company. Interpretations for the characters in the red boxes are presented in fig. S6 [(A) and (B)]. (**C**) Temporal variations in the (upcoming) rain pieces ratio (RR) and the (upcoming) heavy rain pieces ratio (HRR), which are reconstructed through quantitative analyses of oracle bone scripts, and temporal changes in typhoon activities in southwestern Japan and South Korea (marked as dots KI and YR in Fig. 1A). Here, Sr (int) denotes the integrated peak area of the Sr peak. (*23*–*24*). The different widths of the blue bars represent the time coverage of each oracle bone script phase, with the blue dots indicating the same period coverage as the blue bars. (**D**) Typhoons In-Fa and Cempaka and the induced extreme rainfall [data from the Integrated Multi-satellite Retrievals for Global Precipitation Measurement (IMERG)] on 20 July 2021. The vectors denote the wind field at 850 hPa. The red line provides the track of typhoon In-Fa, with the markers indicating the typhoon center location at 12:00 UTC each day. The small picture in the top-left corner is an enlarged view of the topography and rainfall distribution.

Oracle bone scripts, the earliest form of systematic Chinese writing, were mostly found in AY in the Central Plains and generally considered as divination records of royalty and nobility in the Late Shang Dynasty (*14*–*15*, *36*). Therefore, besides being recognized as invaluable cultural relics, oracle bone scripts also serve as precious contemporaneous documentation of people and their circumstances during the Chinese Bronze Age (*37*–*38*). Consistent with that revealed in the relevant geological and archeological records, oracle bone scripts also suggest a considerable influence of climate extremes, especially floods, during the Late Shang Dynasty (*8*, *39*–*41*). A typical example can be seen in Fig. 2A; one of the main characters representing “disaster” in oracle bone scripts is a pattern resembling water waves, which indicates floods according to existing studies (*42*).

Notably, modern disaster research also highlights that floods, especially those triggered by extreme rainfall events, are among the deadliest and most economically damaging meteorological disasters in China, due to both immediate destruction and long-term effects such as waterlogging, soil salinization, and pest outbreaks (*43*–*44*). Therefore, a quantitative analysis of the meteorological information about oracle bone scripts was conducted in this study to reconstruct climate conditions, particularly rainfall variations around 3000 yr B.P. in the Central Plains. Specifically, we examined the rainfall-related contents in ~55,000 pieces of oracle bone scripts (belong to five phases with a ~200-year duration; table S3), which are retrieved from authoritative collections and studies with relatively explicit interpretations (table S4). Through counting the varied proportions of oracle bone script pieces that contained inquiries about “(upcoming) rain” and “(upcoming) heavy rain” in the five oracle bone script phases (Fig. 2B, fig. S6, A and B, table S5, and Supplementary Text), we built up two indexes to describe the variation trend of rainfall situations during the Late Shang Dynasty.

As shown in Fig. 2C, the two reconstructed indexes, the (upcoming) rain pieces ratio (RR) and the (upcoming) heavy rain pieces ratio (HRR), presented marked fluctuations during the five phases. The up and downs of RR, which shows how much the Late Shang people were concerned about the (upcoming) rain, were highly consistent with the well-resolved variations in northward typhoon activities, which were retrieved from typhoon-induced sediments in southwestern Japan (*23*) and South Korea (*24*). What is more, accompanying the most intensified northward typhoon activities in the oracle bone script phase three, HRR presented an extraordinarily higher level than in any other phase. This immediate increase in the Shang society’s concern about the upcoming rain, especially the upcoming heavy rain, during periods of intensified northward typhoon activity, suggested a potentially substantial contribution of northward typhoon to the extreme rainfall events in the Central Plains. These contributions are also evident in modern cases. Historical high-impact extreme rainfall events in the Central Plains, along with the induced floods [e.g., the floods of August 1975, August 1996, and July 2021 (Fig. 2D)] (*33*, *35*, *45*), were always found to be related to northward typhoon activities (*33*).

#### Correlation between westward typhoon and floods in the Chengdu Plain

**Fig. 3.**
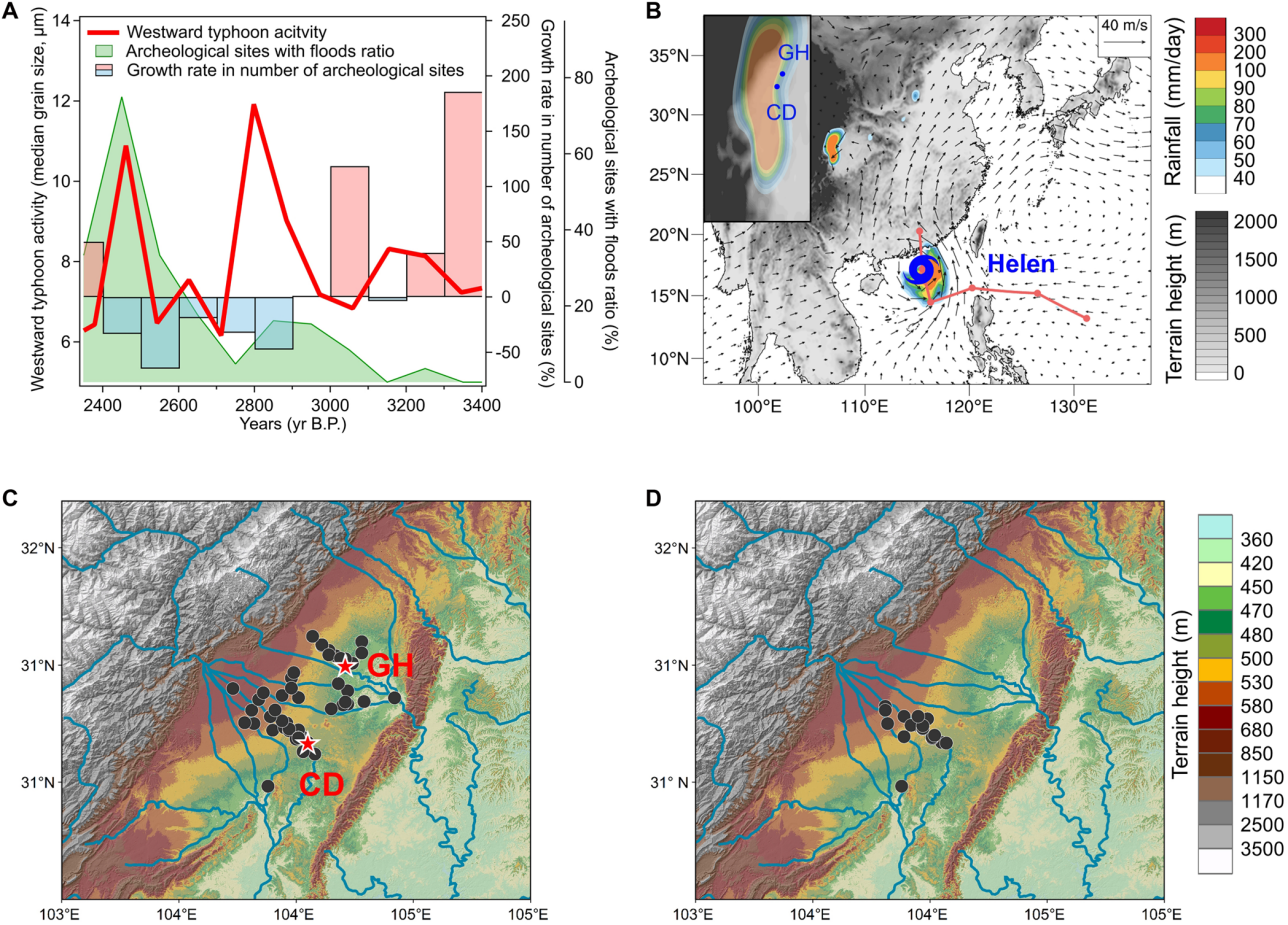
Flood situations recorded in archeological sites in the Chengdu Plain and their correlation with westward typhoon activities. (**A**) Temporal variations in westward typhoon activities (*26*) and ratio of archeological sites with flood layers; growth rate in number of archeological sites in the Chengdu Plain. (**B**) Tropical cyclone Helen induced extreme rainfall [data from the China Meteorological Forcing Dataset (CMFD) dataset] on 11 August 1995. The vectors denote the wind field at 850 hPa. The red line provides the track of tropical cyclone Helen, with the markers indicating the typhoon centers location at 12:00 UTC each day. The small picture in the top-left corner is an enlarged view of the topography and rainfall distribution. (**C** and **D**) Distribution of archeological sites (black dots) during periods of weak (3100 to 3000 yr B.P.) and strong (2800 to 2700 yr B.P.) westward typhoon activities, respectively. The red stars in (C) give the locations of GH and CD.

Similar to the potential impact of northward typhoons on archeological cultures in the Central Plains, the ancient Shu civilization in the Chengdu Plain, as mentioned before, experienced abrupt social changes along with the intensification of westward typhoon activities (*26*) during the mid-late Holocene (Fig. 1B). To look into the relationship between westward typhoons and social changes in the Chengdu Plain, we further analyzed the flood layers in the archeological excavation data here (from 125 archeological sites; table S1 and fig. S2). As shown in Fig. 3A, along with the intensification of westward typhoon activities around 2800 and 2450 yr B.P., flood layers were found in a notably greater proportion of archeological sites in the Chengdu Plain. Meanwhile, in periods characterized by intensified westward typhoon activities, archeological sites not only decreased in numbers but also became geographically concentrated in the relatively elevated areas (Fig. 3, C and D), where people would suffer less under the impact of flood disasters. These evidences, thus, implied a possibly high correlation between westward typhoon activities and Chengdu Plain floods during the mid-late Holocene. Correspondingly, modern cases also illustrate the connection between westward typhoons and flood disasters in the Chengdu Plain, such as the extreme rainfall event around 11 August 1995 (Fig. 3B). Triggered by tropical cyclone Helen reaching the Pearl River Estuary (PR)—the location where the westward typhoon proxy was retrieved and used in this study—the event caused the “95.8” flood disaster, affecting more than 427,000-hectare crops and trapping 310,000 people (table S2) (*46*–*48*).

#### Additional evidence of typhoon-coincident climate extremes

Besides the temporal alignment of intensified typhoon activity with extreme rainfall and flooding, archeological and geological records from the Central Plains and the Chengdu Plain provide additional evidence of flood events and their societal impacts during periods of intensified northward and westward typhoon activity around 3000 yr B.P. (Supplementary Text). For example, in the Central Plains, geological records from the Longmen Gorge (in the Yiluo region) indicate that flood peaks around 3100 to 3000 yr B.P. reached twice the magnitude of the largest observed floods in modern times since 1937 (e.g., the 1958 typhoon-induced event) (*49*), while archeological evidence near ZZ reveals settlement destruction and forced relocations due to flooding during the Late Shang Dynasty (*41*, *50*). In the Chengdu Plain, remains of flood-damaged buildings around 2900 yr B.P. and remains of flood-destroyed dikes around 2450 yr B.P. have been identified, with studies suggesting that flood disasters severely affected the rice-based agriculture of the ancient Shu civilization and potentially altered settlement patterns (*51*–*54*).

In addition, the abundant historical records from around 1000 yr B.P., another period of intensified typhoon activity though less pronounced than around 3000 yr B.P., further support our analysis of the correlation between typhoons and inland floods, as well as their impacts. As shown in fig. S7, flood-drought index in the North China Plain (*55*), including the Central Plains, aligns with the trend of northward typhoon activity, while flood records from the Minjiang and Tuojiang rivers in the Chengdu Plain (*56*) are mostly found in periods with intensified westward typhoon activities. Meanwhile, historical documents provide explicit cases of typhoon-induced severe floods and their disastrous impacts in the study regions (Supplementary Text) (*57*–*58*). Collectively, these records suggest that typhoons may have played a key role in driving inland floods and social changes.

### Mechanisms of typhoon activity affecting inland regions

#### Modern meteorological data statistics

Although the climate around 3000 yr B.P. differs from the modern climate, the overall climatic patterns remain similar (*2*, *59*). Therefore, modern meteorological data can be used to understand the impact of typhoon activity on inland regions to some extent. As shown in fig. S8A, when regional daily rainfall exceeded 50 mm/day, nearly 60% of rainfall events near ZZ and AY (1950 to 2021) were related to typhoon activities. Northward typhoons even contributed more than 85% of the rainy days when regional daily rainfall exceeded 75 mm/day (see Materials and Methods). Similarly, as shown in fig. S9A, westward typhoons could contribute 90% when daily regional rainfall exceeded 75 mm/day. Meanwhile, in the upper-quartile years with the most northward (westward) typhoons from 1950 to 2021 (1979 to 2018), the eastern slopes of Mount TH (Mount HD) suffered from more than 40% (60%) increases in the extreme rainfall days (greater than 75 mm/day) (figs. S8B and S9B). The explanation here could be that typhoons, regardless of whether they make landfall or not, have a probability to induce an easterly wind anomaly, intensifying water vapor transported to the windward slopes of north-to-south mountains, thereby enhancing rainfall in these regions (*33*, *60*–*64*). In addition, the east-to-west mountain ranges located to the south of the Central Plains and the Chengdu Plain can effectively block the warm, humid southeast and southwest monsoons, making extreme rainfall in these two regions very sensitive to typhoon activity (fig. S10).

### AI- and physics-based model simulations

In addition to long-term meteorological data analysis, we seek to quantitatively evaluate the impact of variations in typhoon activity on extreme rainfall in inland regions through model simulations. As the increase in typhoon activity may suggest an overall increase in typhoon intensity (see Materials and Methods and fig. S11) (*65*–*66*), we focus on changes in typhoon intensity in our simulations. Two representative cases of typhoon-induced extreme rainfall were selected and simulated by the Weather Research and Forecasting (WRF) model: one in the Central Plains (1976 case; fig. S12) and the other in the Chengdu Plain (1995 case; fig. S13). These cases were chosen on the basis of their intense extreme rainfall (regional average exceeding 100 mm/day) and the associated flooding, as well as the typhoon tracks passing through southwestern Japan [Kamikoshiki Island (KI) in Fig. 1A] and PR (in Fig. 1A), where the northward and westward typhoon proxy data used in this study were obtained. As shown in figs. S12 and S13, when typhoon intensity decreased, the easterly winds striking the windward slopes of the mountains weakened, leading to a notable reduction in moisture flux convergence (MFC) in the areas around ZZ, AY, and CD (*67*), causing a more than 50% decrease in rainfall in these regions. These results, therefore, suggested that intensified typhoon activities could considerably strengthen the convergence of water vapor and, consequently, add to the extreme rainfalls in the Central Plains and the Chengdu Plain.

**Fig. 4.**
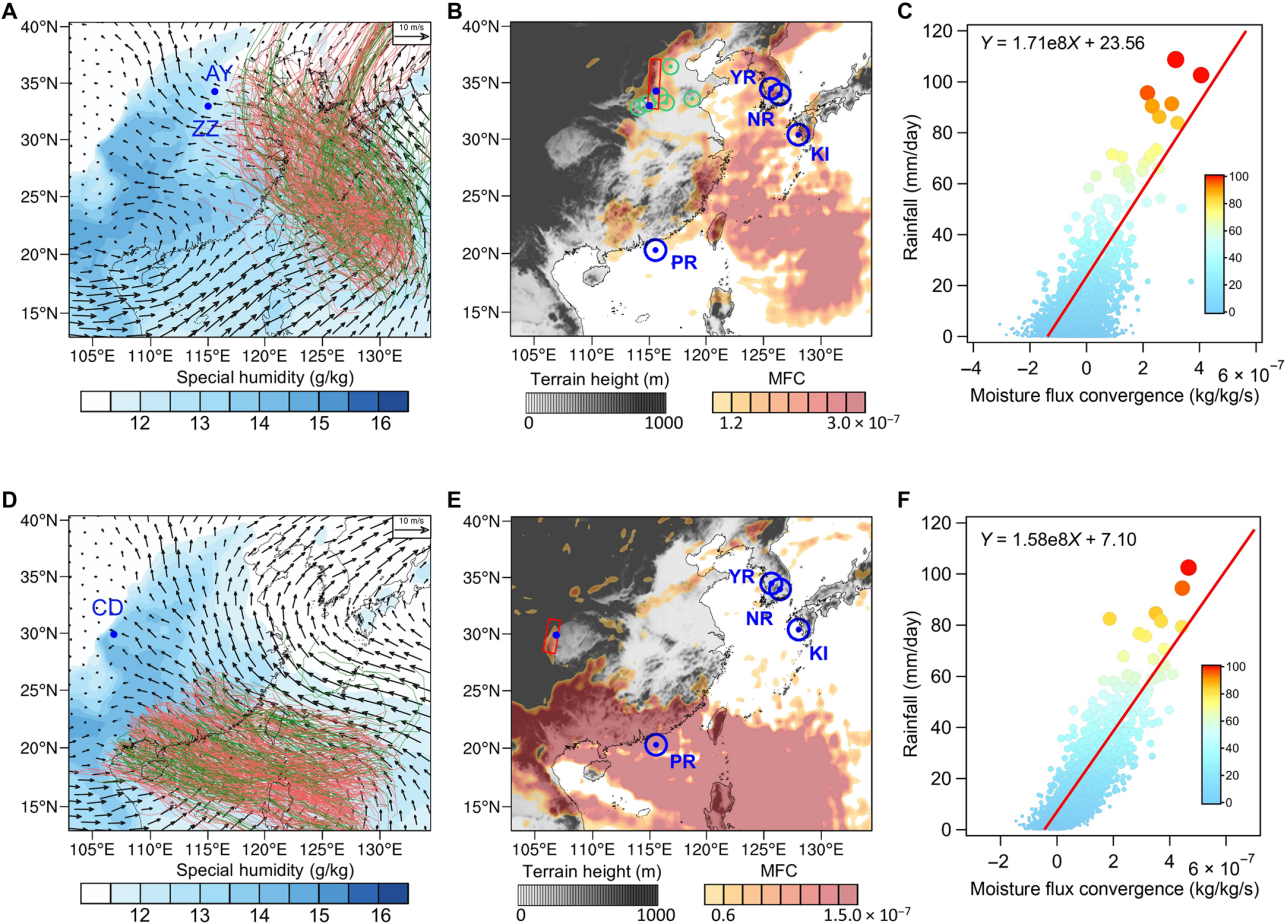
Simulated changes in extreme rainfall due to an increase in typhoon intensity. (**A**) Average specific humidity distribution and wind field at 850 hPa when northward typhoons occur; total of 261 cases. Tracks of weak and strong typhoons are shown in green and red lines. (**B**) Changes of upper quartile of maximum MFC between simulations with strong and weak typhoons. Blue circled dots give the locations where mid-late Holocene typhoon proxies used in this study are retrieved (*23*–*26*). Green circled dots show the distribution of flood deposits that document flood events around 3000 yr B.P. when northward typhoon activities increased (table S6). (**C**) Scatter plots of MFC and daily rainfall in the Central Plains [red box in (B)] in summer time during 1950 to 2021. The red line shows the reduced major axis regression, which was set using the fitting function labeled on the top-left corner. (**D** to **F**) Same as (A) to (C) but for westward typhoons (total of 319 cases), MFC, and daily rainfall in the Chengdu Plain.

Considering the varying impacts of individual typhoons on rainfall in inland regions, this study further combined the AI-based Pangu-Weather model (*16*) with physics-based models [Community Earth System Model version 2 (CESM2) and WRF] to conduct an idealized experiment involving a large number of cases, aiming to quantify the impact of an overall increase in typhoon intensity (similar to the period around 3000 yr B.P.) on extreme rainfall in inland regions. Specifically, we tried changing the typhoon intensity in a fixed climate state in our idealized experiments to explore the impact of intensified typhoon activities (see Materials and Methods and fig. S14). As shown in Fig. 4, an overall increase in typhoon intensity (maximum wind speed of inserted typhoon seeds rising from 15 to 35 m/s) can largely strengthen the maximum MFC over East Asia. For northward typhoons (Fig. 4A), the intensified ones enhanced the moisture transport by the easterlies (fig. S15, A and C), increasing the maximum MFC on the eastern side of the mountains by about 3 × 10^–7^ kg/kg per second on average (Fig. 4B), thus substantially enhancing extreme rainfall by about 51 mm/day in the Central Plains (Fig. 4C). These results can also be proved by the similar distribution of enhanced maximum MFC and mid-late Holocene flood deposits (Fig. 4B), which document flood events around 3000 yr B.P. during a period of intensified northward typhoon activities (table S6). Similarly, for westward typhoons, the increased intensity of typhoons also strengthened the moisture transport toward the Chengdu Plain (Fig. 4D and fig. S15, B and D), leading to a notable increase in the maximum MFC by about 1.5 × 10^–7^ kg/kg per second (Fig. 4E) and thereby increasing extreme rainfall in the Chengdu Plain by about 24 mm/day (Fig. 4F) and causing heavier floods there.

## DISCUSSION

Focusing on climate extremes, this study reveals the impacts of millennial-scale variations in typhoon activities on extreme rainfall, flood disasters, and social changes in inland China around 3000 yr B.P. However, the climate conditions during the mid-late Holocene were highly variable (*2*), and other climatic hazards may have also played substantial roles in shaping societal developments. For instance, our analysis of oracle bone scripts suggests that the varied proportions of oracle bone script pieces that contained inquiries about “pray for rain” and “locust plague,” which serve as proxies for drought severity, closely follow the reconstructed El Niño–like conditions by Moy *et al.* (*68*) (fig. S16). This phenomenon aligns well with the modern meteorological studies, which finds that El Niño events can induce severe droughts in the Central Plains (*69*). Therefore, although the correlation between monsoonal precipitation and population trends appears weak over long timescales (fig. S17), it remains possible that extreme droughts induced by El Niño–like conditions notably influenced societal stability. This pattern bears similarities to the climatic challenges faced by the Maya civilization, where prolonged El Niño–like conditions may reduce overall rainfall while intensified tropical cyclone activity could increase extreme rainfall, ultimately contributing to social declines (*70*).

Previous paleoclimate studies have identified a correlation between intensified northward/westward typhoon activities and increased/decreased El Niño–like conditions around 3000 yr B.P. (*23*, *25*), which can also be found in this study (fig. S18). Notably, this spatial pattern aligns with modern El Niño–Southern Oscillation (ENSO)–typhoon relationship (*71*–*73*). Specifically, recent work shows that El Niño and La Niña events amplify typhoon rainfall in Korea, Japan, and Pearl River Delta (*72*), where the northward and westward typhoon proxy data used in this work were retrieved. However, the typhoon activity can be shaped by multiple factors (*74*–*75*), and the El Niño–like conditions around 3000 yr B.P. were complex, with studies suggesting that this period coincided with a transitional phase in ENSO expression (*59*). Therefore, despite the observed co-occurrence of intensified typhoon activity and ENSO anomalies, the underlying causes of the intensified typhoon activity around 3000 yr B.P. and the extent to which ancient El Niño events resemble their modern counterparts remain unclear and lie beyond the scope of this study.

By integrating archeological radiocarbon database, archeological evidence, oracle bone scripts, and paleo-typhoon proxies, our study first reveals the links between coastal typhoon activity, inland extreme rainfall, flooding, and social changes during 4000 to 2000 yr B.P. On the basis of this, we combine modern atmospheric data analysis and numerical simulations to explain how typhoons induce inland extreme rainfall and floods while also quantifying typhoon’s impacts on the magnitude of rainfall and floods to some extent. Moreover, by integrating records of typhoon-induced floods from both modern times and around 1000 yr B.P. (when typhoon activity also high), our study further illustrates the substantial impact of typhoon-driven floods on inland populations and societies. While our findings are based on a comprehensive synthesis of diverse evidence and detailed analysis, uncertainties remain in two key aspects. First, archeological records and paleoclimate proxies carry inherent chronological uncertainties and representativeness biases. Second, our meteorological analyses rely on modern climatology, which may not fully capture conditions around 3000 yr B.P., including the mean state of large-scale circulation systems such as the western Pacific subtropical high. Variations in these background fields could affect typhoon steering and the resulting inland rainfall, which introduces additional uncertainty (*72*, *76*–*77*). Even so, by integrating multiple lines of proxy evidence, we partially address these limitations and enhance the robustness of our interpretations (see Supplementary Text). Further validation will depend on the future availability of high-resolution paleoclimate proxy records and continued improvements in paleoclimate modeling (*78*).

The debate over mid-late Holocene climate extremes in China, especially their impacts on the social changes during the Bronze Age, has lasted for decades (*7*–*9*). In this study, we demonstrated that intensified typhoon activities made a substantial contribution to climate extremes, population decline, and social changes in the Central Plains and the Chengdu Plain during this period. While the climate-driven social changes during this period remain to be fully understood, our results highlight the necessity of considering typhoon influences on climate extremes in inland areas and assessing their relationship with social development in ancient times. Meanwhile, similar to the reconstructed situations of intensified typhoon activities during the mid-late Holocene, today’s world is also faced with an increasing challenge of typhoon-induced extreme events, owing to the marked climate change nowadays mainly caused by human activities (*73*, *79*–*80*). As revealed in model predictions, a 14% increase in typhoon intensification could occur by 2100, even under a moderate climate change scenario (Representative Concentration Pathway 4.5) (*73*). Considering modern models commonly underestimate the intensity of extreme events (*81*), this study urges better preparation against the disastrous impact of intensified typhoons, especially in inland areas where facilities to mitigate extreme rainfalls and floods are relatively inadequate.

## MATERIALS AND METHODS

### SPD for the Central Plains of China

The SPD of ^14^C data is widely used as a proxy for population size. In this work, SPD values were generated using the spd package in R based on ^14^C dates (149 dates; sampling locations are shown in Fig. 1A as green dots) from Wang *et al.* (*22*) and calibrated with the IntCal20 curve (*82*). In that dataset, multiple ^14^C dates from the same site or context had been combined using OxCal’s R_Combine (*83*) to avoid overrepresentation of intensively dated sites (*22*). In addition, a 300-year moving average was applied to smooth the SPD, reducing artificial peaks and troughs that may arise from nonlinearities in the radiocarbon calibration curve (*22*, *84*).

### Determining the impacts of typhoons on rainfall from reanalysis data

Typhoons can contribute to rainfall in inland China directly (if a typhoon lands inland) or indirectly (by providing favorable meteorological conditions). Therefore, we considered a rainfall event to be a typhoon-influenced one if an accompanying tropical cyclone reached the level of a severe tropical storm and passed through the southeastern seas of China (8°N to 38°N and 105°E to 140°E) on the same day the rainfall was recorded. In addition, we define a northward or westward typhoon–influenced rainfall event in this study based on whether the typhoon’s track reaches north of 28°N. The distribution ranges of typhoons were determined according to multiple typical cases in which typhoons have caused extreme rainfall on the Central Plains and the Chengdu Plain. The typhoon tracks data are from the Collection of the Tropical Cyclones Best-Track Data (CMABSTdata) (*85*).

In this study, we used two distinct rainfall datasets to analyze the rainfall events in the Central Plains and the Chengdu Plain. For the cases in the Central Plains, we used the land component of the fifth generation of European ReAnalysis (ERA5-Land) data, which spans from 1950 to now and has a spatial resolution of 0.1° and a temporal resolution of 1 hour. This dataset was selected for its extensive temporal coverage (*86*). In contrast, for the cases in the Chengdu Plain, we used the China Meteorological Forcing Dataset (CMFD). The CMFD, covering the period from 1979 to 2018, provides high spatial (0.1°) and temporal resolution (3 hours) and is specifically designed for studying land surface processes in China’s complex terrains (*87*). Despite its shorter temporal span, the CMFD was chosen for its accuracy in regions with intricate topography. By integrating these datasets, we leveraged the strengths of each to comprehensively examine regional rainfall patterns.

### Quantifying the impact of typhoon intensity on extreme rainfall in East Asia

#### Typical case simulations with WRF

To quantitatively understand the effects of typhoons on extreme rainfall in the Central Plains and the Chengdu Plain, we performed two parallel numerical experiments using the WRF model. (i) A regular simulation (EXP_strong) was conducted with the meteorological field initialized by European Centre for Medium-Range Weather Forecasts (ECMWF) ReAnalysis version 5 (ERA5) data 1 day before the cases and was run for 72 hours, with the first 24 hours as model spin-up and the last 48 hours for the final analysis. (ii) An idealized numerical simulation, similar to the EXP_strong simulation, was run; in this simulation, the typhoon intensity in the initial meteorological fields was reduced by half (by halving the maximum wind speed of typhoons Helen and Therese) using Bogus (EXP_weak) (*88*). The effects of intensified typhoons on rainfall can be quantified by comparing the difference between EXP_strong and EXP_weak. The applied modeling settings are shown in table S7.

#### Multicase simulations with Pangu-Weather

While the climate around 3000 yr B.P. differed from that of the present day, the dominant typhoon pathways appear to have remained broadly comparable, with most typhoons tracking either northward or westward (*89*). Moreover, the variation in typhoon activity during this period shows a consistent trend with that of sea surface temperature (SST) (fig. S11), and because elevated SSTs are commonly associated with stronger typhoons (*73*), we propose that the increase in typhoon activity was more likely reflected in the overall intensity of typhoons (*89*).

To simulate the impact of changes in typhoon intensity on extreme rainfall in East Asia, we need to vary typhoon intensity while keeping the climate state fixed. Given that typhoons can induce extreme rainfall closely linked to topographic effects, either through direct landfall or interactions with the large-scale background circulation, simulations require large domains and high spatiotemporal resolution. In addition, a large number of cases must be simulated to comprehensively represent typhoon activities, posing challenges to our computational resources and methodologies. The Pangu-Weather model (*16*) offers an alternative approach to address this issue. Trained on ERA5 reanalysis data, Pangu-Weather can accurately simulate global circulation, typhoon activities, and topographic effects on circulation at a resolution of 0.25° with high speed.

In this study, we used daily simulation outputs for July and August from the CESM2, with atmospheric chemistry represented by the Whole Atmosphere Community Climate Model version 6, to provide initial meteorological conditions for the Pangu-Weather model. The CESM2 configuration consists of 72 vertical layers, extending up to ~150 km, and a horizontal resolution of 0.95° × 1.25°. The CESM2 simulation covered a 20-year period, of which the first 10 years were treated as spin-up and the remaining 10 years were used for analysis. Initial conditions for the CESM2 simulation were based on a 2010 climatology, calculated as the mean over 2006 to 2014. The model setup follows that of Lu *et al.* (*90*). CESM2’s low spatial resolution allows it to provide meteorological fields with negligible typhoon activity (*91*). Subsequently, a fixed-intensity typhoon seed, which was made by WRF, was inserted into Pangu-Weather model’s initial meteorological fields to generate typhoon activities. The typhoon seed was inserted in the area with the highest probability of typhoon occurrence, which can be seen in Fig. 1A (130°E and 10°N to 17°N and 132°E and 18°N to 25°N, inserting a typhoon at each latitude). Each simulation ran for 7 days at a temporal resolution of 6 hours, with one typhoon seed inserted. Only the cases with the inserted vortex that grows in the environment were used for further analysis in this work.

To simulate the influence of enhanced typhoon intensities on extreme rainfall, we performed three parallel numerical experiments using different fixed-intensity typhoon seeds. Specifically, the maximum wind speed of the inserted fixed-intensity typhoon seed is set to 15, 25, and 35 m/s at the radius of 110 km to represent a weak, normal, and strong typhoon, respectively (fig. S14). As a result, the impacts of typhoon intensity changes on extreme rainfall in our study regions can be quantified by comparing the maximum MFC in each grid between simulations with different typhoon intensity seeds, considering the strong correlation between rainfall and MFC in the Central Plains and the Chengdu Plain (Fig. 4, C and F).

Because of the lack of reliable 3000 yr B.P. climate data, our simulation method cannot authentically reproduce the actual changes in typhoon activity during the 4000–2500 yr B.P. period. However, this limitation does not diminish the value of our research. Instead, it serves as an idealized experiment to explore the potential impact of typhoon activity changes on inland extreme rainfall, providing valuable insights despite the constraints.

## Supplementary Materials

### The PDF file includes:

Supplementary Text Figs. S1 to S18 Tables S1 to S8 Legend for data S1 References
