# Supplementary material for: Archeological data with AI- and physics-based modeling explain typhoon-induced disasters in inland China around 3000 yr B.P
Source: Sci Adv. 2026 Mar 4;12(10):eaeb1598. doi: 10.1126/sciadv.aeb1598 (PMC12959396; doi:10.1126/sciadv.aeb1598)
Supplement: Supplementary file 1 — Supplementary Text Figs. S1 to S18 Tables S1 to S8 Legend for data S1 References [file sciadv.aeb1598_sm.pdf]

Science Advances
AAAS

Supplementary Materials for

Archeological data with AI- and physics-based modeling explain typhoon-induced disasters in inland China around 3000 yr B.P.

Ke Ding *et al.*

Corresponding author: Quansheng Ge, geqs@igsnrr.ac.cn

Sci. Adv. **12**, eaeb1598 (2026)
DOI: 10.1126/sciadv.aeb1598

The PDF file includes:

Supplementary Text
Figs. S1 to S18
Tables S1 to S8
Legend for data S1
References

Other Supplementary Material for this manuscript includes the following:

Data S1

Supplementary Text

Relationships between typhoon activity, flood disasters, and societal consequences

1. Flood evidence during periods of intensified typhoon activities around 3000 BP

Geological evidence

In the Central Plains of China (referred to below as the Central Plains), flood reconstructions from sedimentary records indicate that the Longmen Gorge (in the Yiluo region) experienced large-scale flooding around 3100–3000 BP (*49*). Peak discharges during this event were estimated to be nearly twice as high as those of the largest historically recorded floods since 1937, including the 1958 typhoon-induced flood.

Archaeological evidence

Excavations in both the Central Plains and the Chengdu Plain provide archaeological support for widespread flood events during periods of intensified typhoon activity. In the Central Plains, sites near Zhengzhou have revealed the abandoned villages after being flooded several times, and flood-damaged granaries dating to the Late Shang period (*41, 50*), consistent with a phase of enhanced typhoon activity. Historical records from the Luoyang area (within the Yiluo region) document flood disasters during the Western and Eastern Zhou periods, most of which occurred during intervals of intensified northward typhoon activities (*32, 93*) (see red circled dots in Fig. S5A).

In parallel, archaeological remains in the Chengdu Plain show evidence of severe flood impacts, including flood-damaged architectural structures at the Shi'erqiao site dating to the early Western Zhou period (around 2900 BP) (*51, 52*), and a repeatedly breached dike at the Fangchijie site from the late Spring and Autumn to early Warring States period (around 2450 BP) (*53*). These historical periods align with phases of intensified westward typhoon activity. In addition, existing studies further proposed that flood events likely disrupted rice agriculture in the ancient Shu civilization and may have contributed to shifts in settlement patterns (*54*).

2. Social changes associated with intensified typhoon activity around 3000 BP

From a general perspective, it can be seen from Fig. S5B that overall population decline, along with profound episodes of social changes in the Central Plain always occurred when northward typhoon activity intensified. These changes include significant cultural and dynastic transitions—from Late Longshan to Erlitou, Erlitou to Erligang, and Erligang to Yinxu cultures, as well as from the Western to Eastern Zhou dynasties and from the Spring and Autumn to the Warring States period (Episodes ①–⑤ in Fig. S5A) (*7, 14, 27–31*). Notably, Episode ③ also coincides with five documented relocations of the Shang capital, which historical records suggest may be related to flooding (*32*). While in the Chengdu Plain, some researchers figured out that the sacrificial pits at Sanxingdui were possibly burial sites for failed sacred objects, which may have resulted from ineffective flood control efforts (*94*). Meanwhile, even a fundamental shift in the power structure may have emerged during this period of intensified westward typhoon activity (around 3000 BP), in which secular kingship overtook the authority of ritual-based theocracy (*95–97*).

Furthermore, population size changes at a more localized scale offer clearer evidence of the societal impacts of intensified northward typhoon activity. As shown in Fig. S5B, the population sizes of the Yiluo region (*7*) and Dahecun site (*98*) generally mirror that of the Central Plains, with significant declines coinciding with periods of increased typhoon incursions (black arrows

in Fig. S5B). This trend is most evident around 3550 BP, when a sudden intensification of northward typhoon activity (near Episode ②) was accompanied by notable reductions in both population size and settlement hierarchy in the Yiluo region (*7*). The correspondence between these social changes, as evidenced by archaeological and historical records, and the SPD-based population fluctuations provides a reasonable basis for exploring the possible influence of typhoon activity on societal development.

3. Relationship between typhoon activities and flood disasters around 1000 BP

To explore the relationship between typhoon activity and flood disasters in the Central Plains and Chengdu Plain, we analyzed flood-drought index from the North China Plain (*55*) and obtained historical flood records from the Minjiang and Tuojiang rivers in the Chengdu Plain (*56*) around 1000 BP, another period with intensified typhoon activities (Fig. S3). These datasets were compared with the time series of northward and westward typhoon activities. As shown in Fig. S7, the increase in flooding in the North China Plain (including the Central Plains) corresponds with the intensification of northward typhoon activity, whereas flood records from the Chengdu Plain are primarily concentrated during periods of strengthened westward typhoon activity.

4. Typical cases of inland flood disasters related to typhoons around 1000 BP

To explore more detailed evidence of typhoon-induced inland floods and their disastrous impacts, we referred to the relevant historical documents around 1000 BP, when proxy data present similar intensified typhoon activities (Fig. S3). Typical cases are illustrated as follows:

Case 1: 726 CE (1224 BP) (*57, 58*)

On July 3rd, 726 CE (traditional Chinese calendar), a typhoon struck Lianyungang City (coastal regions), Jiangsu Province. On July 8th, flooding occurred in Mengjin and Jiaozuo (near Anyang), Henan Province in the Central Plains. Then, from July 14 to 18, torrential rains affected several areas in Henan, including Zhengzhou, Anyang, Xinxiang, Hebi, Kaifeng, and Puyang. Riverbanks collapsed and widespread flooding ensued. Thousands of people died, farmlands were severely damaged, crops were completely destroyed, and extensive property losses were reported. Some records even described that “people all lived in trees” to escape the flood.

Case 2: 1077 CE (873 BP) (*57, 58, 99*)

In July 1077 CE (traditional Chinese calendar), a typhoon struck Wenzhou in Zhejiang Province (coastal regions), while severe flooding affected multiple areas in Henan and Hebei Provinces, including regions around Anyang and Handan (in the Central Plains). Floodwaters breached city walls, reached depths of up to 6.6 meters, destroyed granaries and houses, and caused numerous fatalities. In Henan, major levee failures led to a course shift of the Yellow River, affecting approximately 45 counties and over 20,000 km^2 of farmland (for comparison, the 1975 Typhoon Nina flood affected over 10,000 km^2 and displaced more than 10 million people). Within three months after the disaster, emergency grain relief alone (excluding post-disaster projects such as dike reconstruction) exceeded the total tax relief granted in 1075 CE to the Liangzhe region (which corresponds to present-day Zhejiang Province, southern Jiangsu, and Shanghai) in response to a major drought.

Socially and culturally, the flood had lasting impacts. Su Shi, a prominent litterateur in Chinese history, then governor of Xuzhou, led a three-month flood defense that protected the city. In 1078 CE, the people of Xuzhou built the "Yellow Pavilion" on the east gate to commemorate the effort, named for the symbolic triumph of earth over water. At its completion on the Double Ninth Festival, Su Shi hosted a literary gathering and composed poems such as *Composed at the Yellow Pavilion on the Ninth Day*, which vividly describe the flood's destruction and the people's resilience.

Case 3: 1390 CE (560 BP) (*57, 58*)

On August 11th, 1390 CE (traditional Chinese calendar), a typhoon struck eastern China, affecting Shanghai, Jiangsu, and Zhejiang Provinces (coastal regions). On the following day, a major levee failure occurred in Kaifeng (near Zhengzhou), Henan Province in the Central Plains of China. In response to the disaster, the central government provided emergency relief to 15,713 households. A total of 25,020 tales of silver was allocated for this purpose—an amount equivalent to the value of approximately 40,000 tons of rice, based on historical price estimates.

<u>Meteorological information from oracle bones scripts</u>

1. Chronological framework and periodization criteria of oracle bone scripts

The prevailing framework for periodizing oracle bone scripts is the "Five Phases" of oracle bone scripts (*14, 92, 100*), in which each phase corresponds to the reign period(s) of one or more kings during the Late Shang dynasty. The absolute dates of these royal reigns are determined by integrating textual and archaeological sources with methods from history, archaeology, and archaeoastronomy. Key sources include the *Records of the Grand Historian* (*Shiji*), the ancient *Bamboo Annals* (*Zhushujinian*), the *Book of Documents* (*Shangshu*), archaeological findings from Yinxu, oracle bone scripts, and Western Zhou bronze texts. This interdisciplinary approach has yielded a relatively robust chronological consensus (*14*).

In recent years, technological advances such as radiocarbon (^{14}C) dating have enabled direct dating of oracle bone scripts. Approximately 100 pieces of oracle bones have now been analyzed using this method, and the overall results align closely with the "Five Phases" chronology established through historical and archaeological reconstruction. This agreement further substantiates the reliability of the "Five Phases" framework in establishing the absolute chronology of oracle bone scripts (*14*).

2. Statistical reliability of meteorological information in oracle bone scripts

Oracle bone scripts, as divinatory records used by the royal family and nobility of the Shang dynasty, were concerned with predicting future events rather than documenting those that had already occurred (*15, 36–38*). Each inscription typically consists of four parts: the introductory statement (noting the date and the person conducting the divination), the charge statement (posing the question or issue of concern), the divinatory statement (the diviner's interpretation or prediction), and the verification statement (a note on whether the predicted event actually happened) (*37*). Only oracle bones containing three key components—the charge, divinatory, and verification statements—can provide definitive information on whether the predicted event actually occurred. For instance, the charge and divinatory statements might ask, "Will it rain tonight?" with a corresponding verification stating either "It did rain tonight" or "It did not rain tonight." However, such complete inscriptions are extremely rare and unevenly distributed across the "Five Phases", making them unsuitable for statistical analysis (see Table S8).

Notably, despite being divinations rather than direct records, the oracle bone scripts, due to their large quantity and temporal continuity, offer a statistically meaningful dataset that provides a more robust basis for quantitative analysis than the limited number of direct records of extreme events. Because divination was deeply embedded in the sociopolitical fabric of Shang society (*15, 36*), with nearly all important affairs subjected to such consultations, temporal variations in the frequency of divinations on specific topics, such as rain, can serve as proxies for changing societal concerns and the importance of those topics.

Building on this rationale, we interpret variations in the proportion of divinatory statements concerning "upcoming rain," "heavy rain," and "pray for rainfall" within each of the Five Phases of oracle bone scripts as reflections of how rainfall, extreme rainfall, and droughts were perceived during different periods. A higher proportion of such divinations in a given phase suggests heightened concern among Shang communities regarding hydrometeorological events and their potential impacts on daily life. Accordingly, significant shifts in these proportions across phases can serve as effective proxies for changes in the frequency and societal impact of extreme weather events, providing a valuable historical dataset for exploring past climate variability and its potential drivers.

Assessment of the representativeness of paleo-typhoon proxies used in this study

The time series of northward typhoon activity used in this study is derived from the averaged and normalized sediment-based proxy records from four sites: two located in Korea (NR and YR) (*24, 25*) and two in Japan (NKI5 and KI2, both within the KI region) (*23*). As shown in Fig. S3, these records exhibit broadly consistent trends, with prominent peaks in typhoon activity around 3000 BP and 1000 BP. Notably, the higher-resolution series from YR and NKI5 show strong agreement not only in the timing of major peaks but also in finer-scale variations (Fig. 2C). Despite an uncertainty of ±35–50 years in most ^{14}C dating, the consistent patterns among the proxies support the combined series as a representative reconstruction of northward typhoon activity.

For westward typhoon activity, the sediment-based paleo-typhoon proxy from the PR region was adopted (*26*). Additional records in South China Sea, such as those from Hainan (*101*), which cover the time span of our study, exhibit broadly similar patterns to the PR record, with shared peaks at ~3900 BP, 3600 BP, 3200 BP, and 2500 BP (Fig. S4). While this general agreement supports the regional relevance of the PR record, some differences exist—for example, the 2800 BP peak observed in PR is absent in the Hainan data. Such discrepancies likely reflect variations in sensitivity of typhoon proxies to typhoon tracks. Given its more southerly and westerly position relative to PR, Hainan is likely more responsive to typhoons that follow more southward or westward tracks. In contrast, the PR site may be better positioned to reflect typhoons that impact the inland Chengdu Plain. This is supported by CMFD data, which show that the most intense typhoon induced extreme rainfall event in the Chengdu Plain over the past four decades (1979–2018) was associated with a typhoon that passed through the PR region (Fig. 3B). Overall, the PR record serves as a reliable indicator of westward typhoon activity in this study.

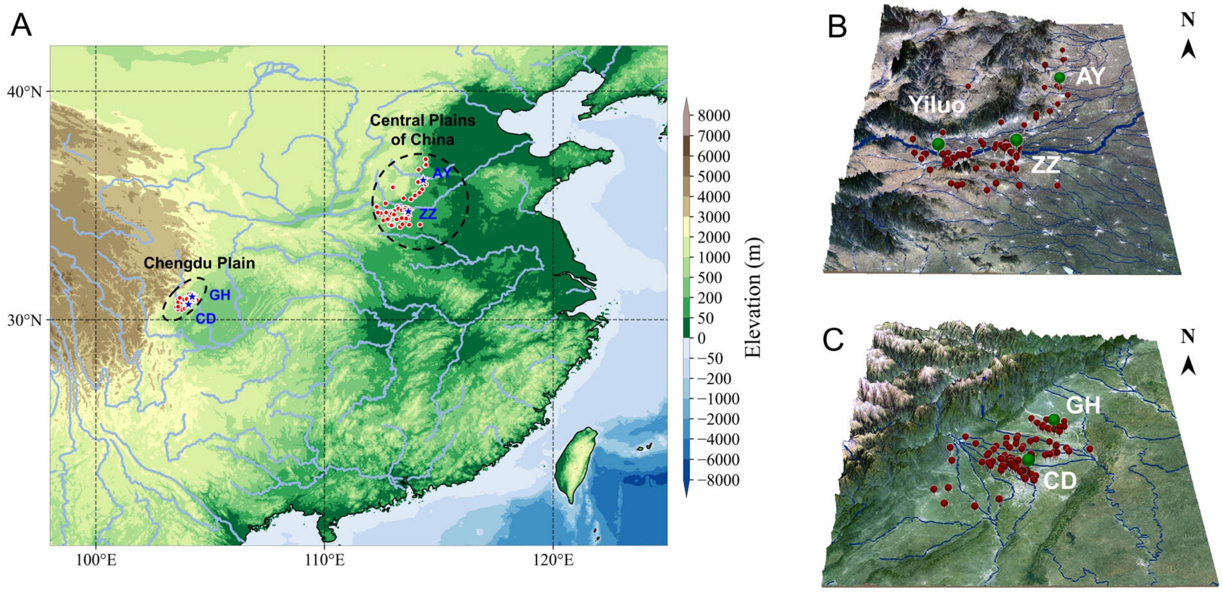

Fig. S1. The distribution of archaeological sites and topography in the Central Plains and the Chengdu Plain. (**A**) The topography and archaeological sites used in this work. (**B**) Three-dimensional topographic map, location of ^{14}C dated sites (*22*) and river distribution of the Central Plains. (**C**) Same as (B) but for location of archaeological sites in the Chengdu Plain (Table S1).

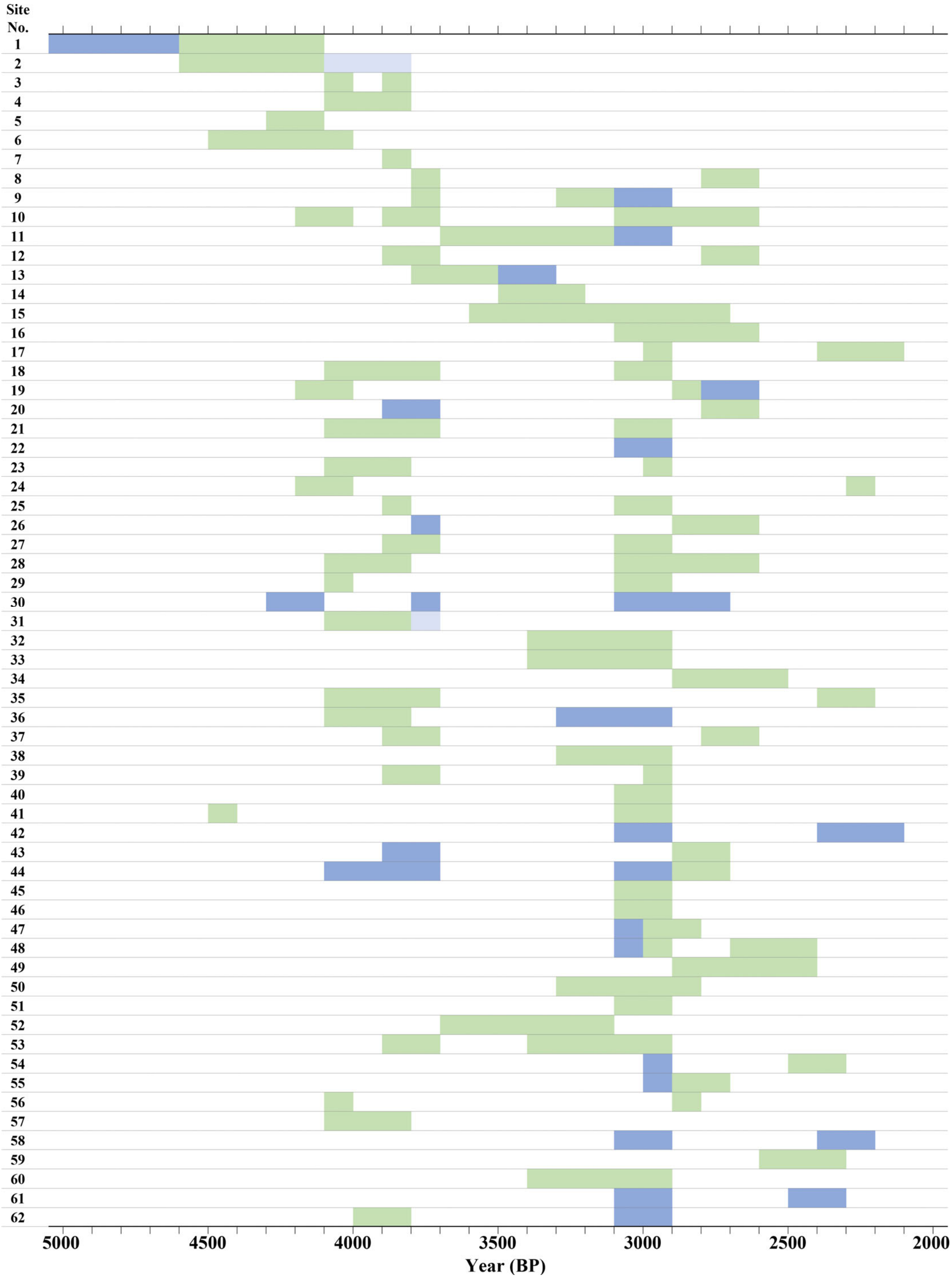

Fig. S2. Temporal distribution of archaeological sites in the Chengdu Plain. Each Site No. corresponds to the same Site No. listed in Table S1. The colored stripes indicate the chronological span of each archaeological site: green represents cultural layers, blue indicates flood layers containing cultural remains, and light blue denotes flood layers without cultural remains. References of the archaeological data for sites in the Chengdu Plain are in Data S1.

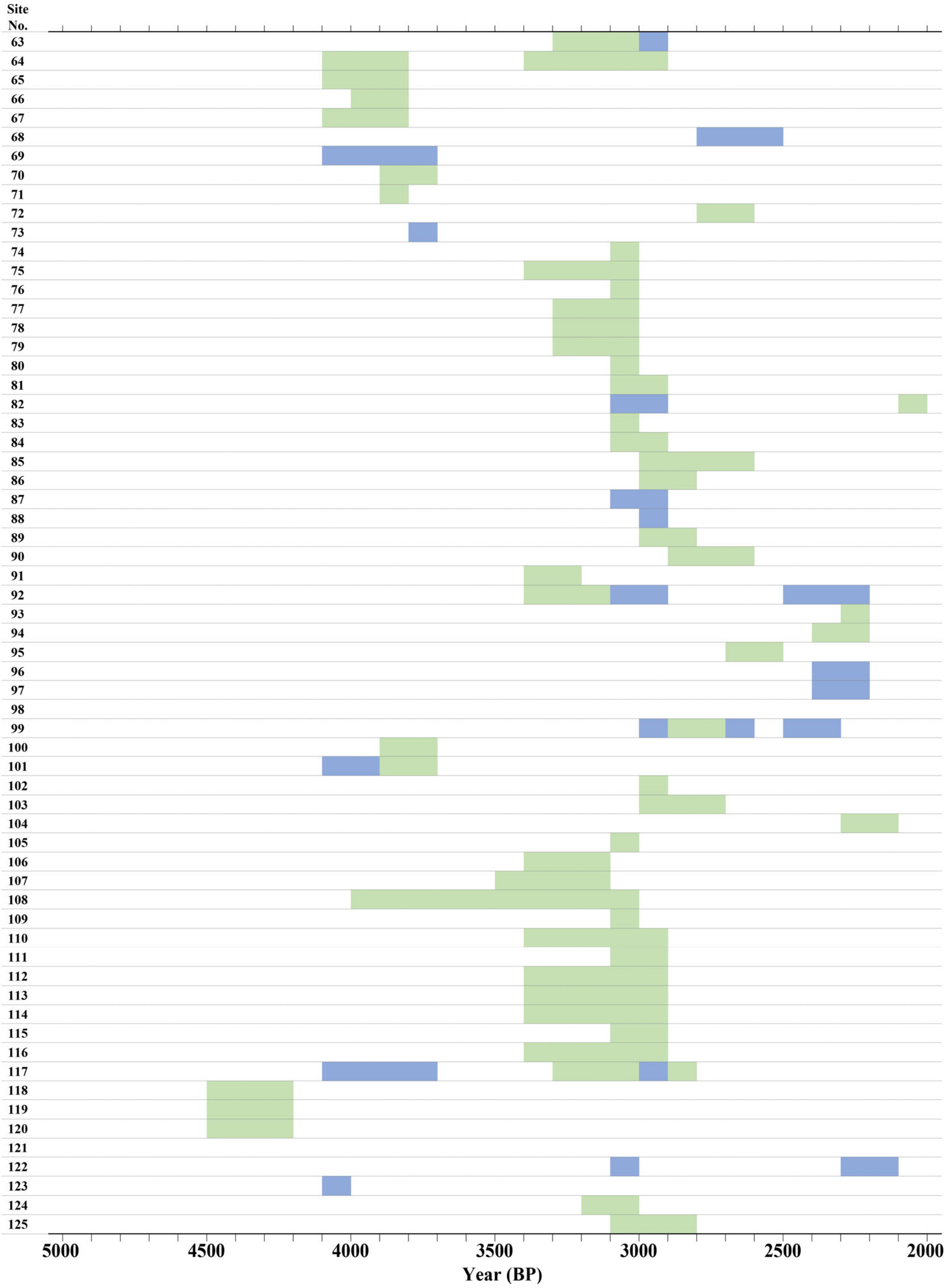

Fig. S2 (continued)

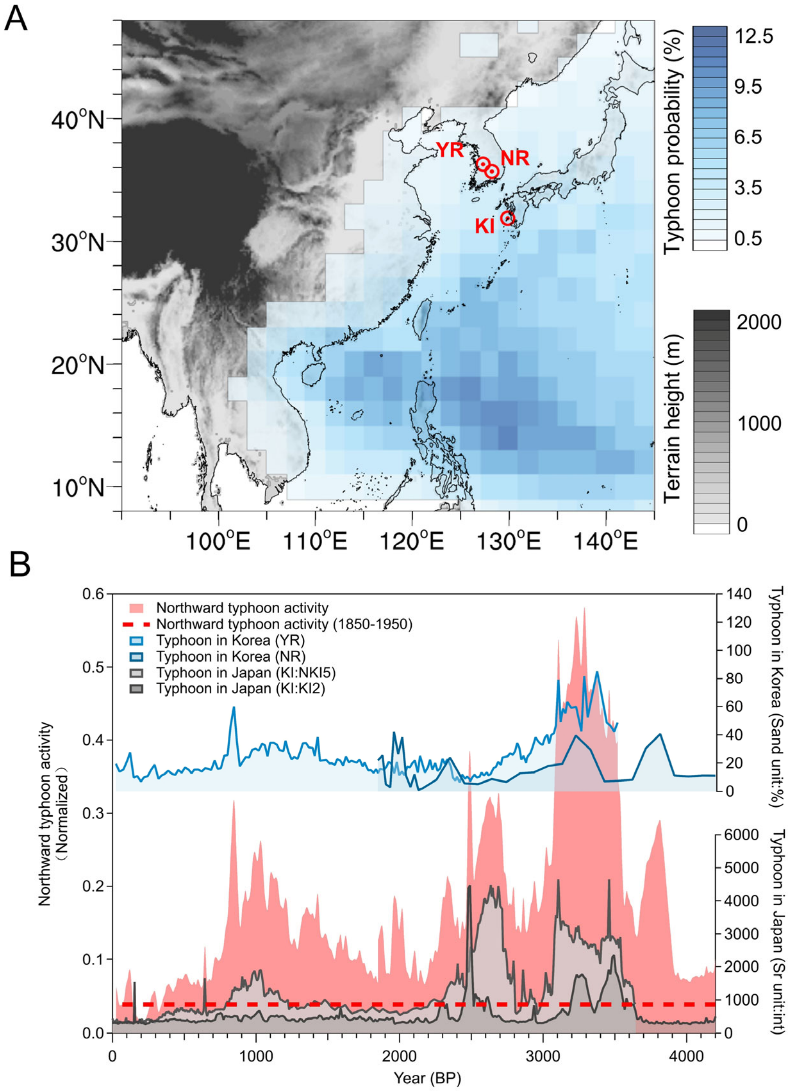

Fig. S3. Locations of paleo-typhoon proxies and northward typhoon activity. (**A**) Distribution of typhoon probability in modern times (1950–2021) and the locations of paleo-typhoon proxies. The paleo-typhoon proxies in YR, NR and KI give the signal of northward typhoon activity (*23*–*25*). (**B**) Northward typhoon activity and its data sources. The northward typhoon activities were normalized from the typhoon proxies got in YR, NR and KI. The red dashed lines give the northward average typhoon activities during 1850–1950.

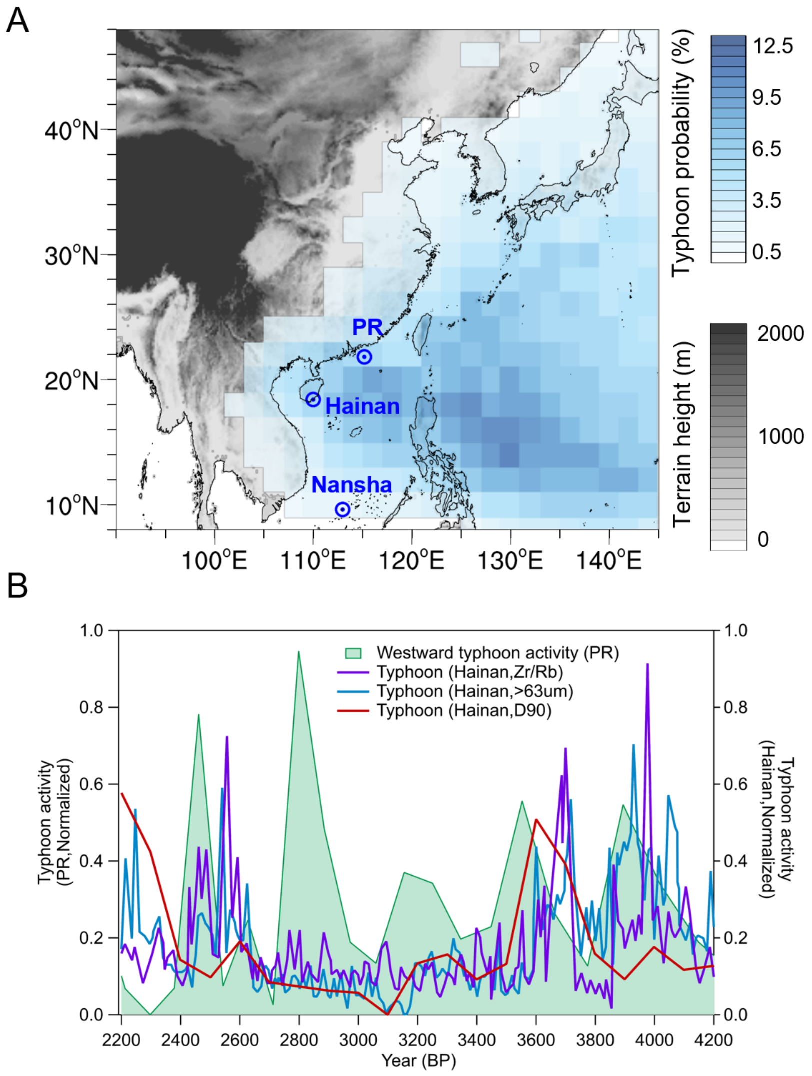

Fig. S4. Locations of paleo-typhoon proxies and westward typhoon activity. (**A**) Distribution of typhoon probability in modern times (1950–2021) and the locations of paleo-typhoon proxies. The paleo-typhoon proxies in PR and Hainan give the signal of westward typhoon activity (*26, 101*). (**B**) Reconstructed variations in typhoon activities in PR and Hainan.

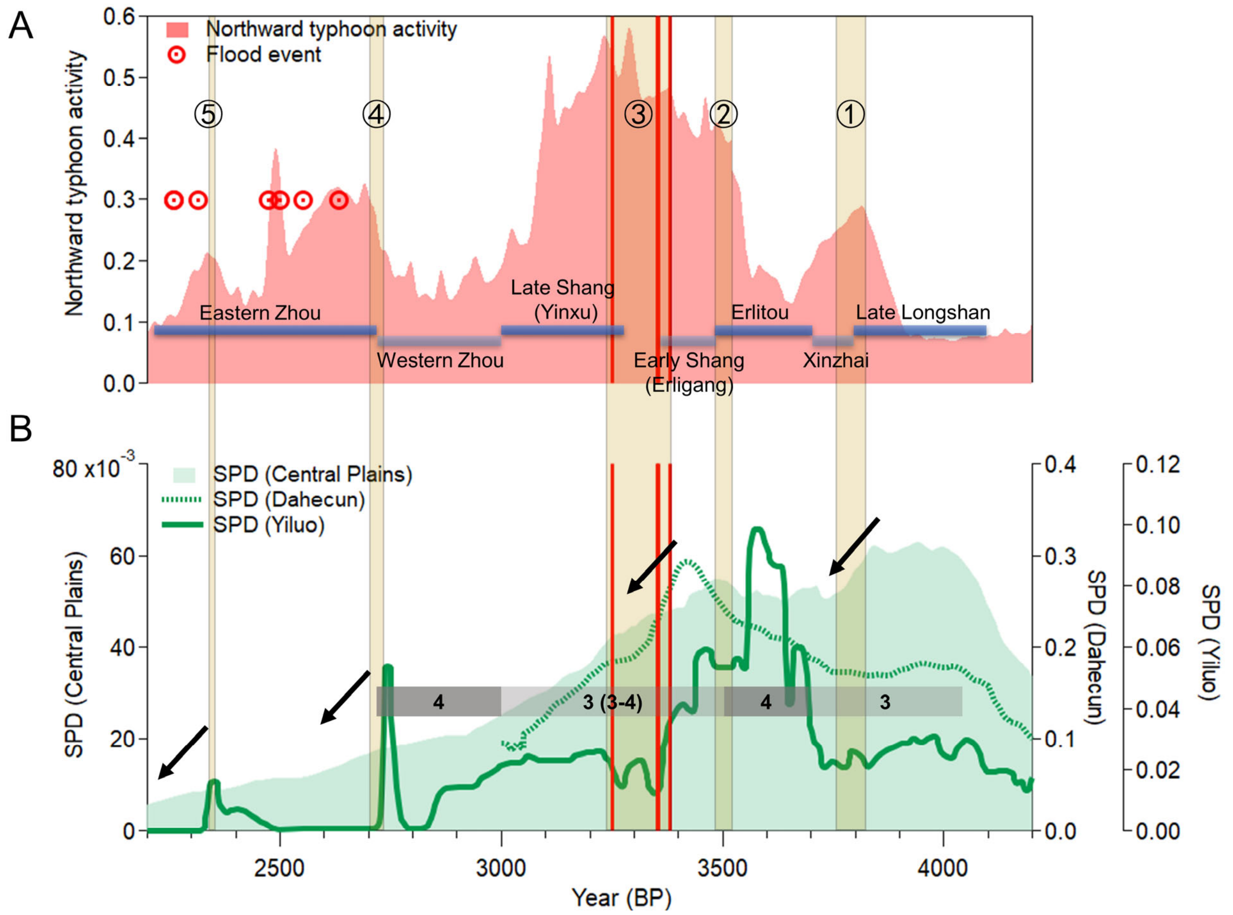

Fig. S5. Temporal correlation between typhoon activity, flood disasters, population size, and social changes. (**A**) Northward typhoon activity, flood records and archaeological cultures and dynasties. Dark and light blue stripes indicate the timespan of archaeological cultures and dynasties (*7, 14*). Red circled dots indicate documented flood disasters in Luoyang (within the Yiluo region) during the Western and Eastern Zhou periods (*32, 93*). (**B**) Population size represented by SPD in the Central Plains (this study), in the Yiluo region (*7*) and at Dahecun site (*98*). The black arrows show significant declines of population size. The grey stripes denote the settlement hierarchy (3 or 4 levels) in the Yiluo region, where more levels reflect greater societal complexity (*7*). The yellow stripes give the profound episodes of social changes (*7, 14, 27–31*), specifically: ① Transition from Late Longshan to Erlitou Culture; ② Transition from Erlitou to Erligang Culture; ③ Transition from Erligang to Yinxu Culture, concurrent with the five relocations of the Shang Capital; ④ Transition from Western Zhou to Eastern Zhou; ⑤ Transition from the Spring and Autumn to Warring States Period. The red lines give the timing of the five documented relocations of the Shang capital (*14, 32*).

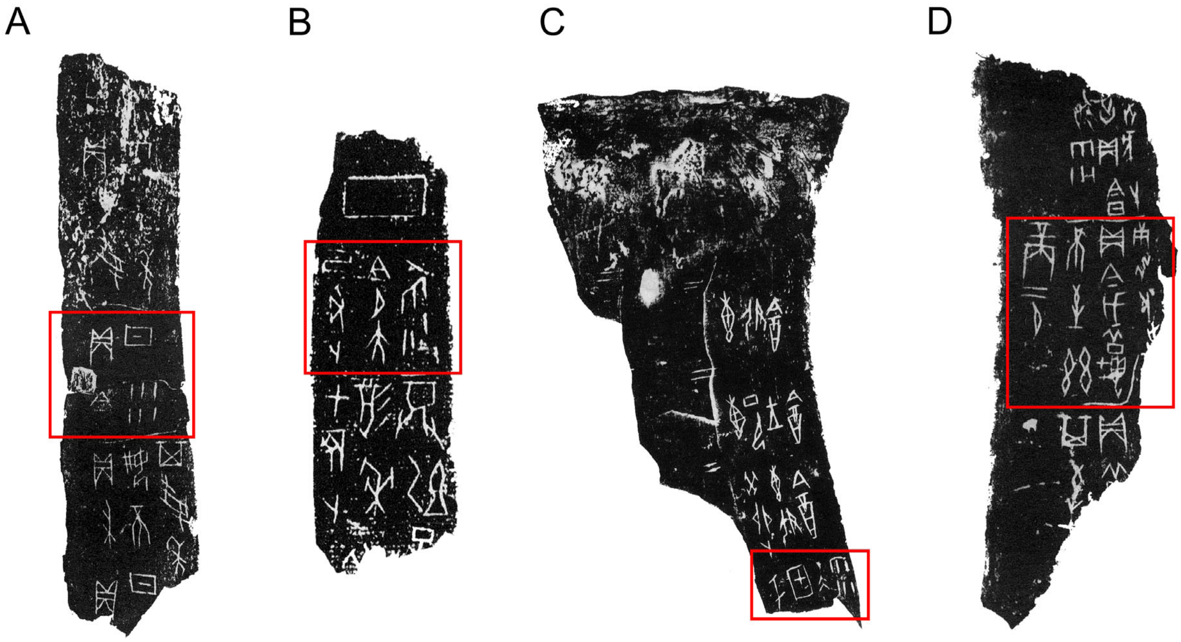

Fig. S6. Typical rubbing examples that contain "(upcoming) rain", "(upcoming) heavy rain", "praying for rain" and "locust plague" (from *The Complete Collection of Oracle Bone Inscriptions* (*92*)). Interpretations of the inscriptions in the red boxes are based on *Interpretation of the Complete Collection of Oracle Bone Inscriptions* (Table S4). (**A**) He 9757, "Divination: Will it rain today?" (**B**) He 27219, "Divination in the day of Jichou: Will there be heavy rain tonight?" (**C**) He 32345, "Shall we pray to (the ancestor) Shangjia for rain?" (**D**) He 24225, "Divination by Chu in the day of Jichou: Will locust plague hit the region of Shang this year?". (A–D) are images reproduced from Guo Moruo (ed.), *The Complete Collection of Oracle Bone Inscriptions* (*92*), used with permission of Zhonghua Book Company.

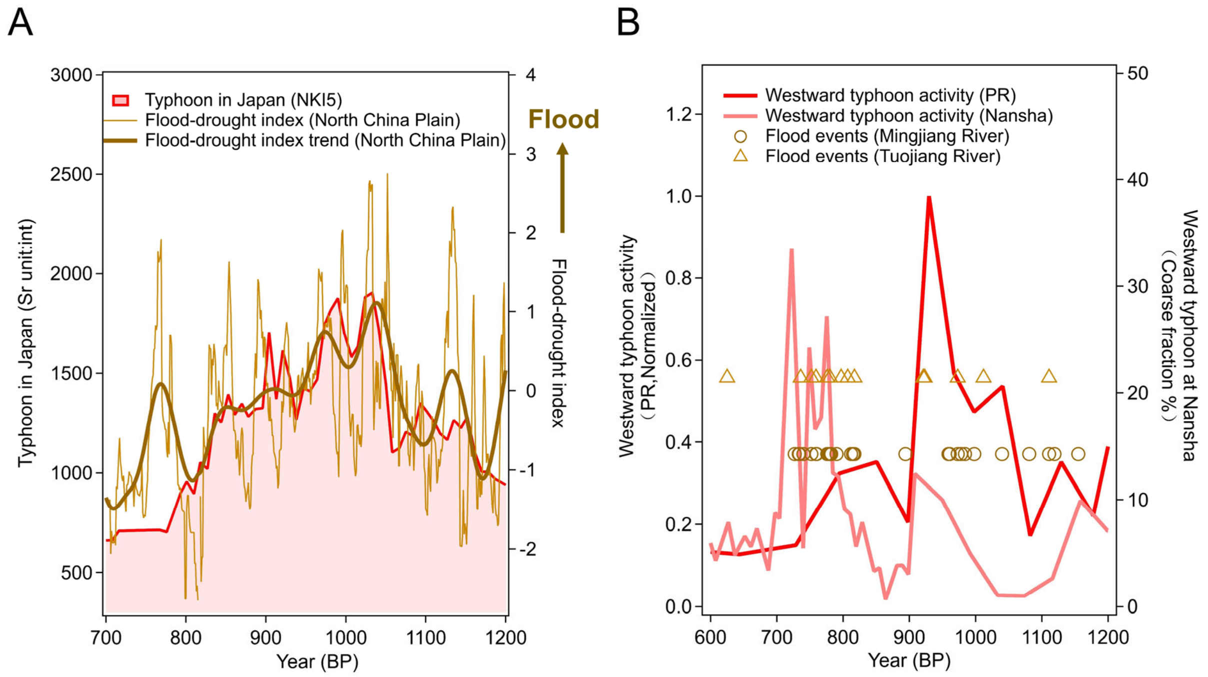

Fig. S7. Relationship between typhoon activities and flood disasters around 1000 BP. (**A**) Reconstructed northward typhoon activities in Japan (NKI5) (*23*) and reconstructed flood-drought index in the North China Plain (*55*) around 1000 BP. The solid brown line indicates the flood-drought index trend extracted using ensemble empirical mode decomposition (EEMD) filtering with a time-scale threshold of over 100 years. (**B**) Reconstructed westward typhoon activities in PR and Nansha (*26, 102*), along with historical records of flood events of Minjiang River and Tuojiang River in the Chengdu Plain (*56*). The locations of PR and Nansha are shown in Fig. S4.

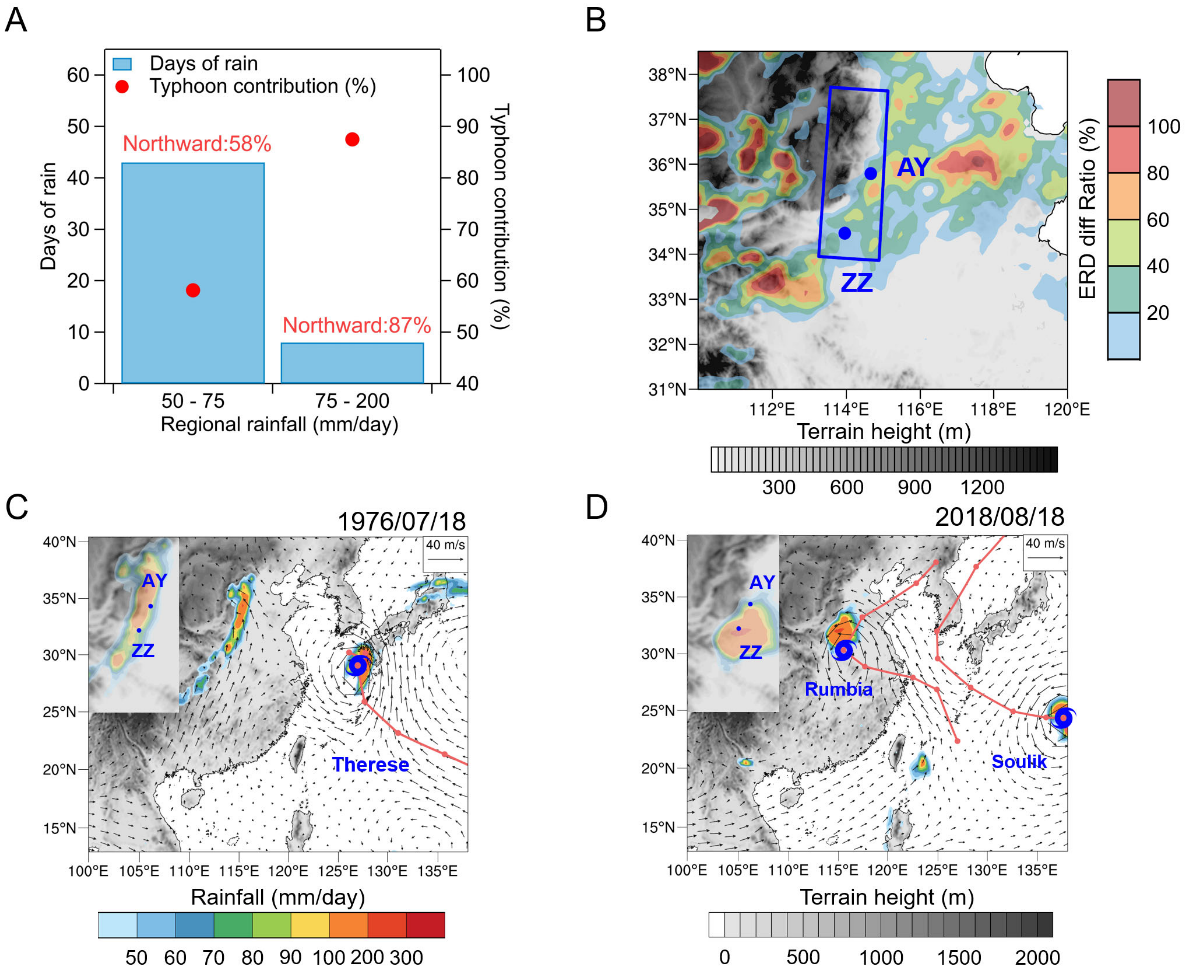

Fig. S8. The relationship between typhoons and extreme rainfall in the Central Plains from 1950 to 2021 based on ERA5_land data. (**A**) The impact of typhoons on the regional daily rainfall in the Central Plains (blue box in (B)) at different rainfall levels. The blue bars indicate the number of days when the regional daily rainfall occurred at different levels from 1950 to 2021 (from ERA5_land dataset), while the red dot shows the contribution rate of typhoons. The contribution of northward typhoons to rainfall is denoted as "Northward". (**B**) Increased ratio of the extreme rainfall day (ERD, daily rainfall in every grid exceeds 75mm/day) number in the upper-quartile years with the most northward typhoons. (**C–D**) Two typical cases whose rainfall exceeded 100 mm/day in Zhengzhou (ZZ) and Anyang (AY) with typhoons passed through southwestern Japan (KI shown in Fig. 1A) (*23*) and South Korea (YR, NR in Fig. 1A) (*24*).

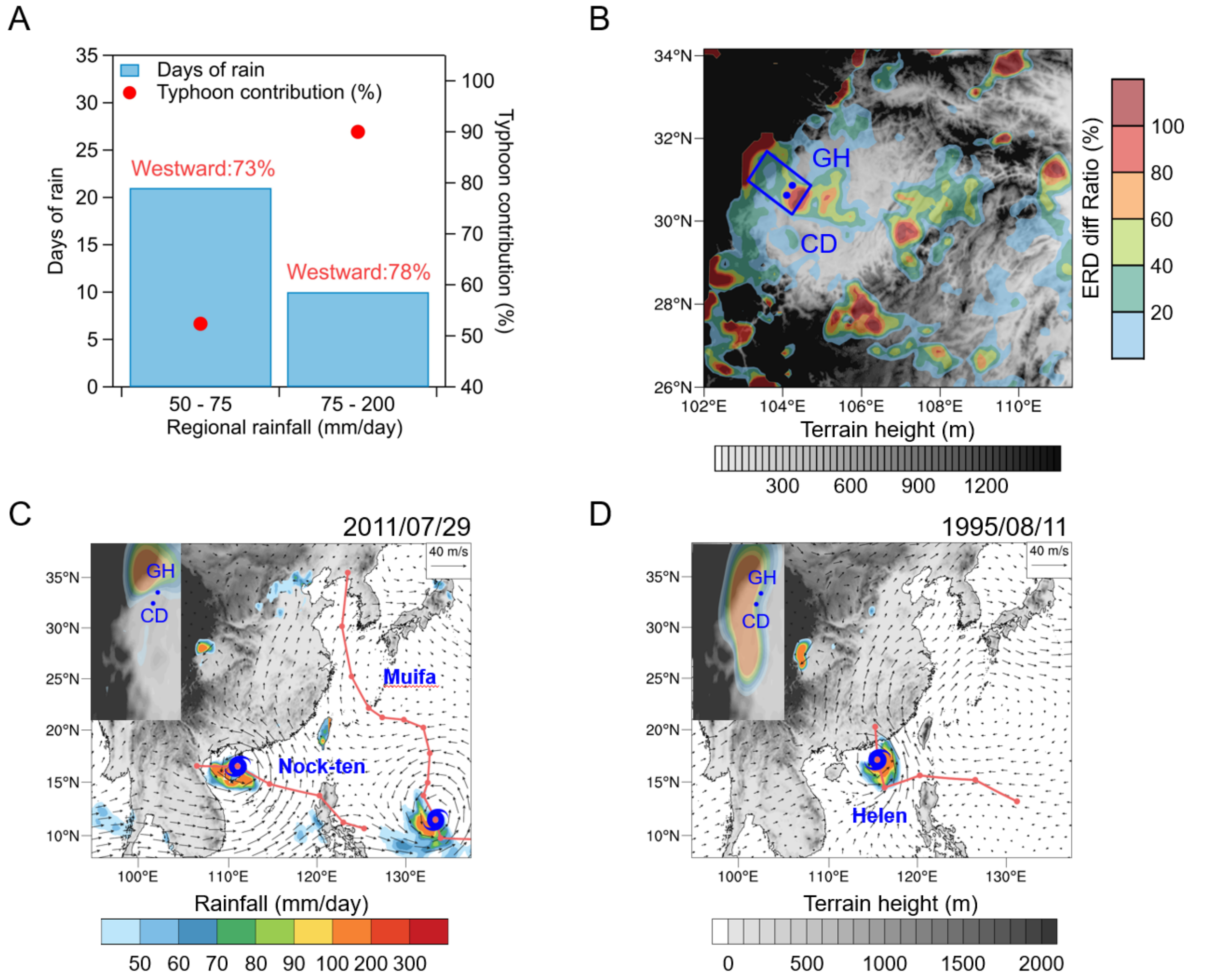

Fig. S9. The relationship between typhoons and extreme precipitation in the Chengdu Plain from 1979 to 2018 based on CMFD and ERA5 dataset. (**A**) The impact of typhoons on the regional daily rainfall in the Chengdu Plain (blue box in (B)) at different rainfall levels. The blue bars indicate the number of days when the regional daily precipitation occurred at different levels from 1979 to 2018 (from CMFD dataset), while the red dot shows the contribution rate of typhoons. The contribution of westward typhoons to rainfall is denoted as "Westward". (**B**) Increased ratio of the extreme rainfall day (ERD, daily rainfall in every grid exceeds 75mm/day) number in the upper-quartile years with the most westward typhoons. (**C–D**) Two typical cases whose rainfall exceeded 100 mm/day in the Chengdu Plain with typhoons passed through PR and Hainan (Fig. S4) (*26*).

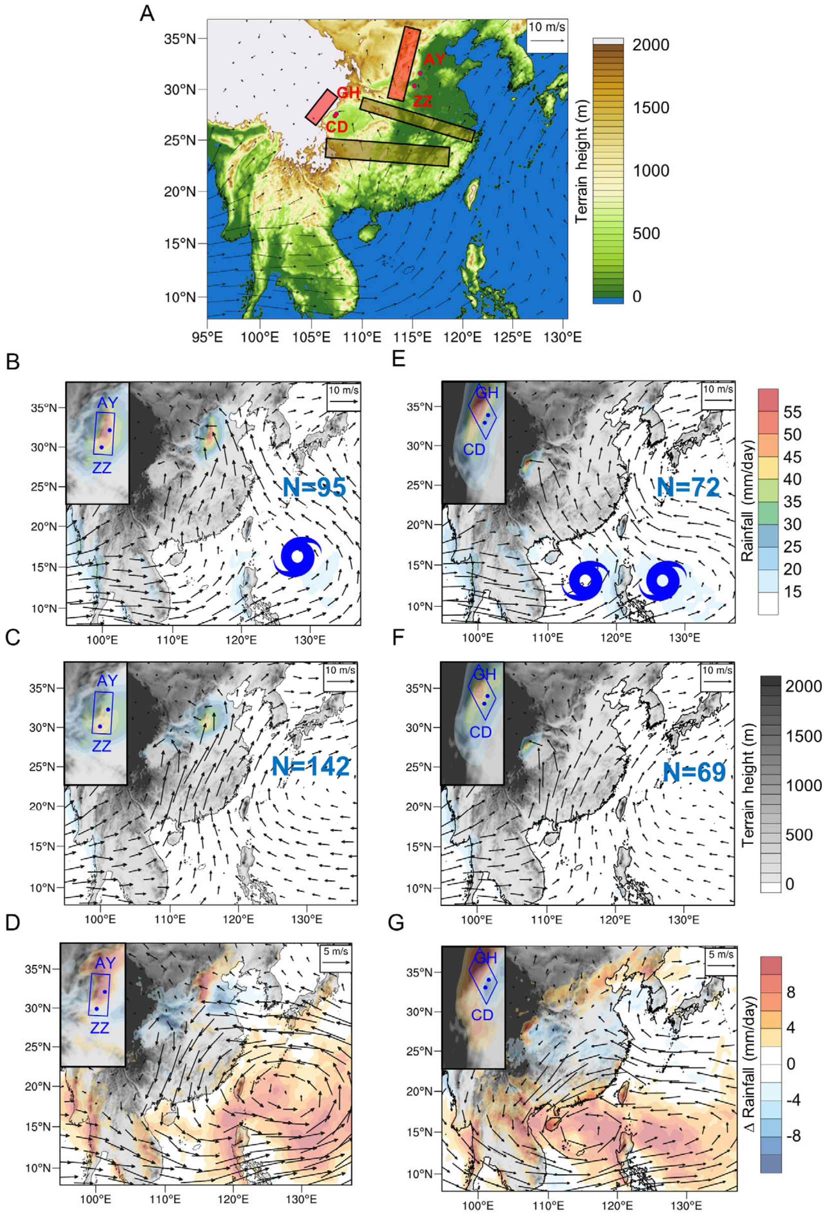

Fig. S10. Distinct types of extreme rainfall in the Central Plains and the Chengdu Plain. (**A**) Terrain and the climatological mean 850-hPa wind field during June–August for the period 1950–2021 (ERA5). Yellow bands indicate east–west–oriented mountain ranges, and red bands denote north–south–oriented ranges. (**B**) Composite mean rainfall and 850-hPa wind fields for cases with daily rainfall ≥25 mm in the Central Plains (blue box) associated with northward typhoons, derived from ERA5_land and ERA5 data for 1950–2021. The small picture in the upper-left corner is an enlarged view of the topography and rainfall distribution. The number indicates sample size. (**C**) Same as (B), but for cases not associated with typhoons. (**D**) Difference in rainfall and wind fields between (B) and (C). (**E–G**) Same as (B–D), but for cases in the Chengdu Plain associated with westward typhoons, using CMFD rainfall data for the period 1979–2018. The blue boxes are same with those in Fig. S8B and Fig. S9B.

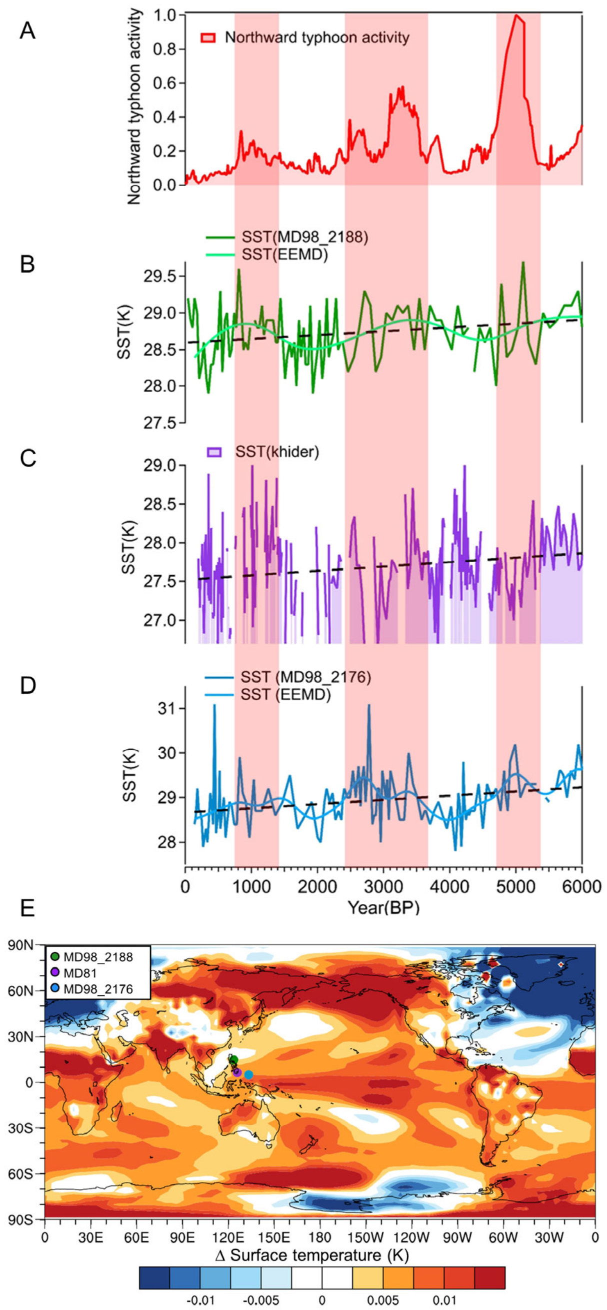

Fig. S11. Time series of northward typhoon activity and corresponding sea surface temperature (SST) records in the western Pacific. (**A**) Intensity of northward typhoon activity. (**B**) SST variations at site MD98-2188 (*103*); the light green line shows the trend extracted by EEMD filtering. (**C**) SST variations at site MD81 (*104*). (**D**) SST variations at site MD98-2176 (*105*); the light blue line shows the EEMD-filtered trend. In (B–D), black dashed lines indicate the linear SST trends. Red bands mark periods of intensified northward typhoon activity. (**E**) Surface temperature anomalies during intensified typhoon periods (3500–2500 BP) relative to background periods (4500–1500 BP), with the data from work of Erb et al. (2022) (*106*). Dots indicate the locations of SST records shown in (B–D).

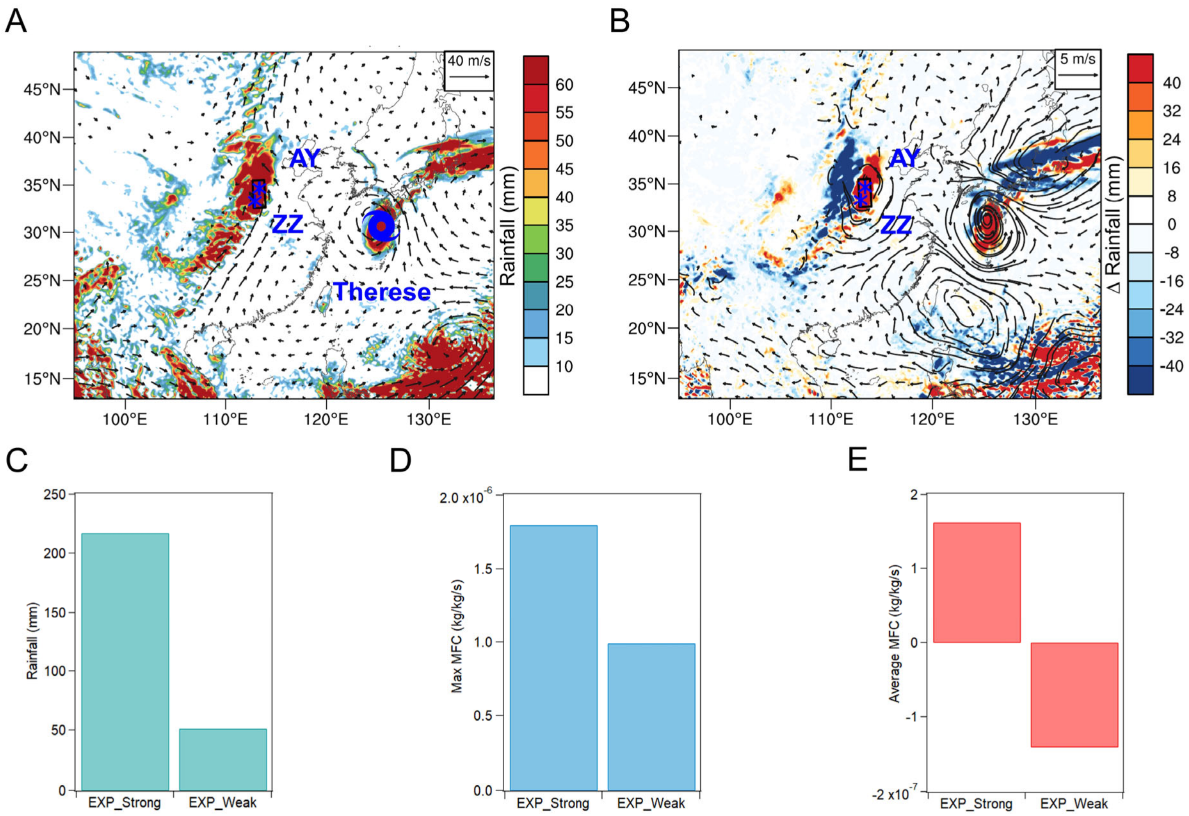

Fig. S12. The influence of northward typhoon Therese on extreme rainfall in the Central Plains simulated by WRF. (**A**) The total rainfall from July 18–19th, 1976, simulated by WRF, with the wind field at 850 hPa. (**B**) Change in rainfall and wind field between simulations with the original and weakened typhoon Therese. (**C–E**) Changes in rainfall, maximum moisture flux convergence (MFC), and average MFC in the Central Plains (black box in (B)) between simulations with the raw (EXP_Strong) and weakened (EXP_Weak) typhoon Therese. ZZ and AY was indicated as blue stars.

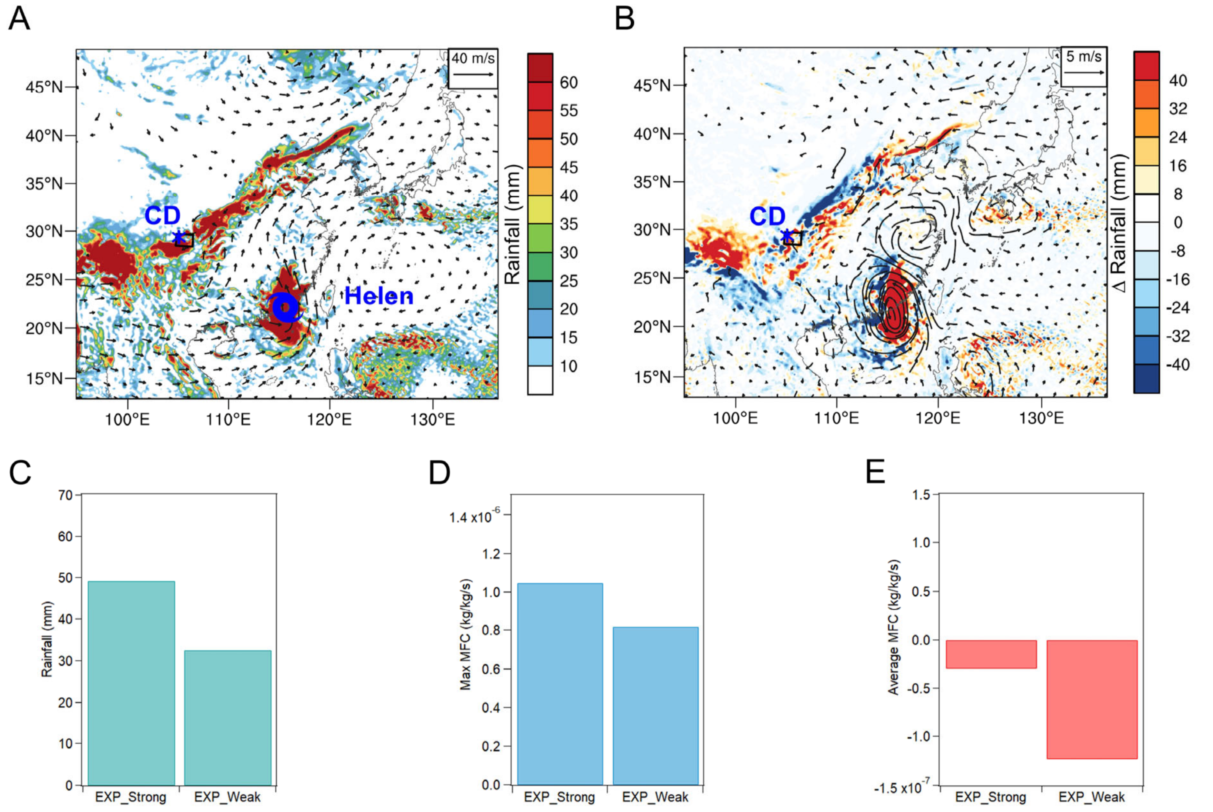

Fig. S13. Influence of westward tropical cyclone Helen on extreme rainfall in the Chengdu Plain simulated by WRF. (**A**) The total rainfall from August 11–12th, 1995, simulated by WRF, with wind fields at 850 hPa. (**B**) Change in rainfall and wind fields between simulations with the original and weakened tropical cyclone Helen (severe tropical storm). (**C–E**) Changes in rainfall, maximum moisture flux convergence (MFC), and average MFC in the Chengdu Plain (black box in (B)) between simulations with the original (EXP_Strong) and weakened (EXP_Weak) tropical cyclone Helen. CD was indicated as a blue star.

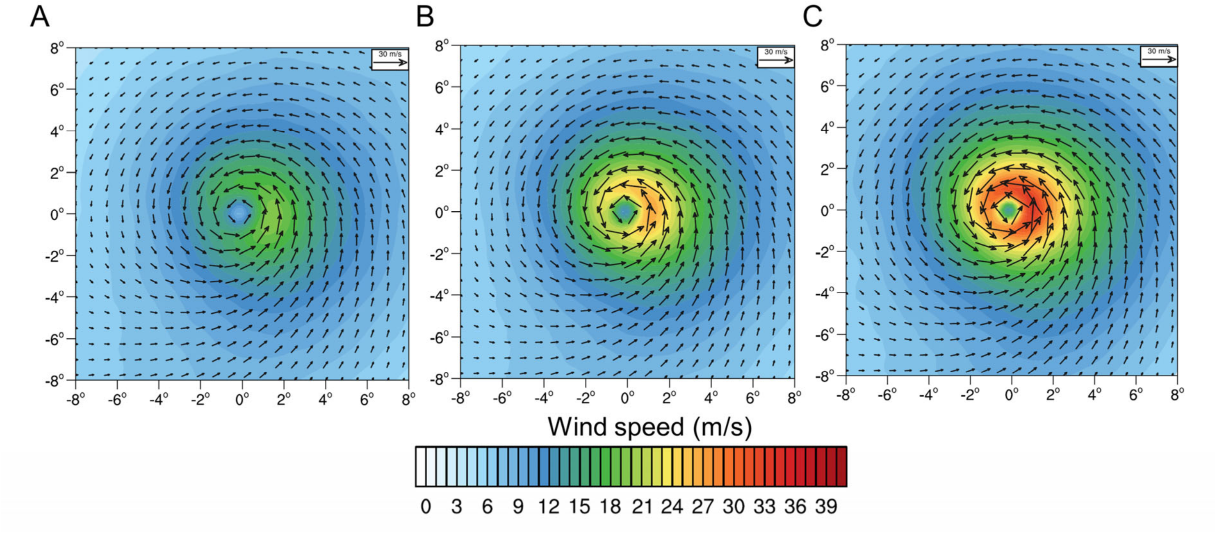

Fig. S14. Wind fields of tropical cyclones with different intensities in ERA5 data from 2000 to 2021. (**A**) Severe tropical storm (observed maximum 2-minute mean wind speed at the surface of 24.5–32.6 m/s) wind field at 925 hPa in ERA5 data. (**B–C**) Similar to (A), but for typhoon and strong typhoon (observed maximum 2-minute mean wind speeds at the surface of 32.7–41.4 m/s and 41.5–50.9 m/s, respectively). The wind fields of tropical cyclones were extracted from ERA5 data based on typhoon eye positions and intensity classifications provided in the CMABST dataset.

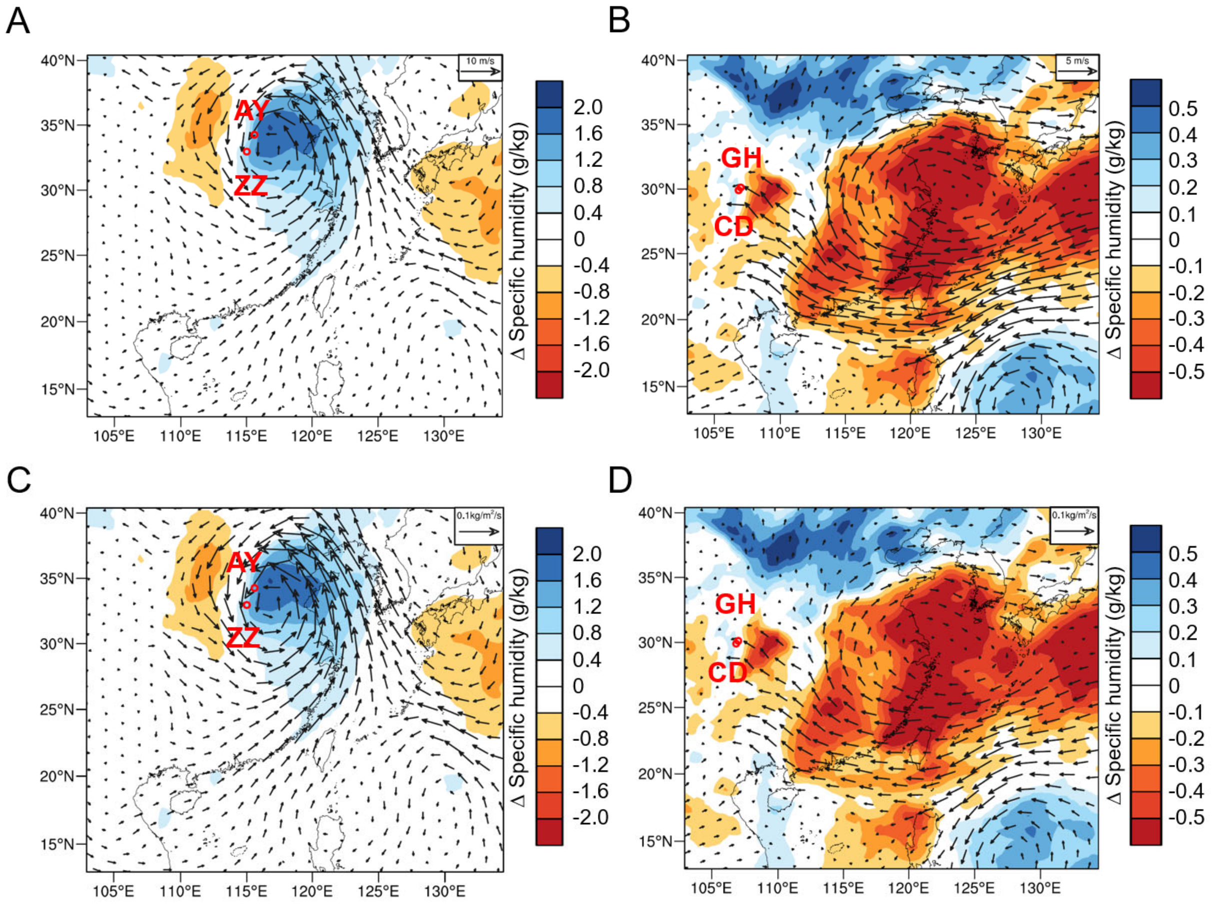

Fig. S15. Impact of intensified northward and westward typhoons on water vapor transport to the Central Plains and the Chengdu Plain. (**A**) Changes in composited specific humidity and wind fields at 850 hPa when moisture flux convergence (MFC) increases over the Central Plains (Fig. 4B red box) due to enhanced northward typhoons. (**B**) Similar changes as in (A), but for the Chengdu Plain (Fig. 4E red box) influenced by intensified westward typhoons. (**C–D**) Same as (A–B) but for moisture flux.

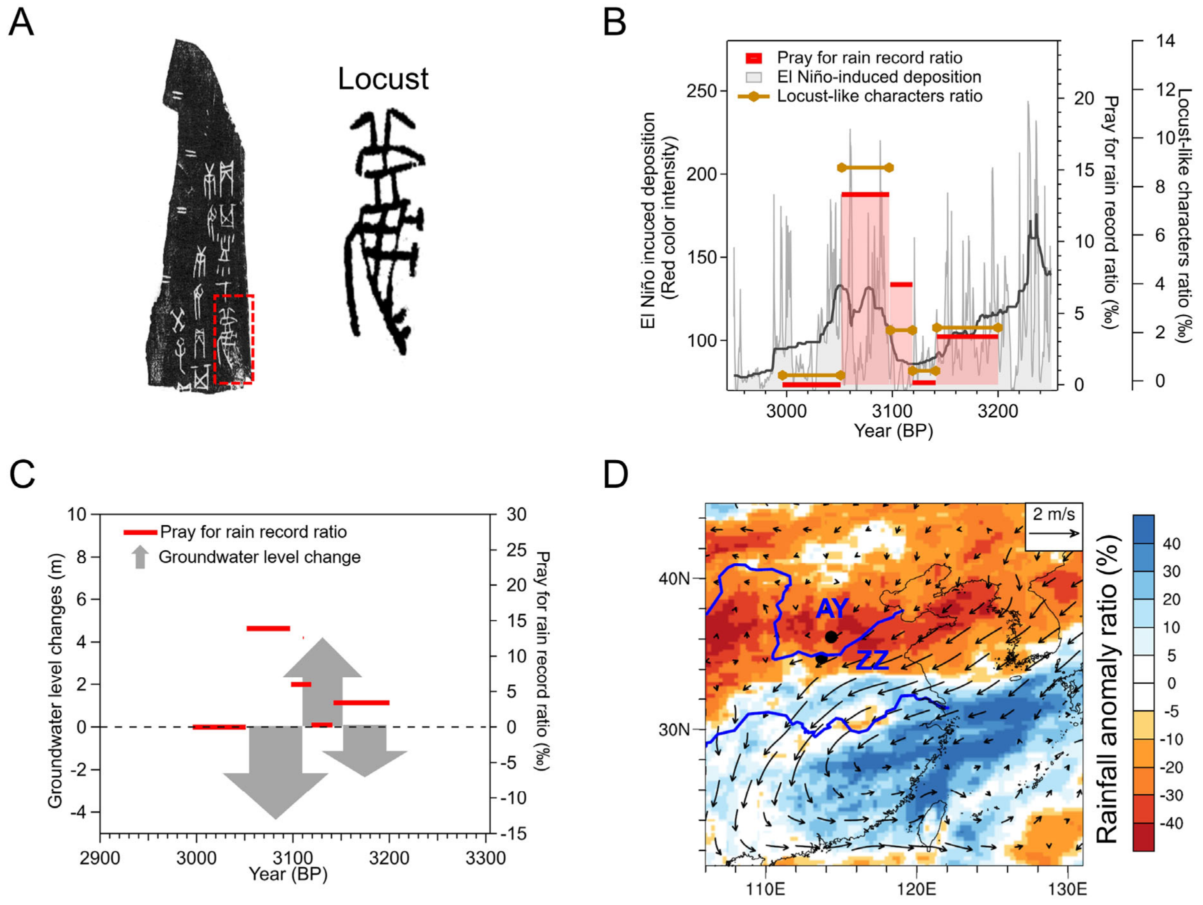

Fig. S16. Rainfall shortage, drought situations in the Late Shang dynasty and El Niño impacts on rainfalls seen in meteorological observations. (**A**) Rubbing of oracle bone script piece (He 33234; image reproduced from Guo Moruo (ed.), *The Complete Collection of Oracle Bone Inscriptions* (*92*), used with permission of Zhonghua Book Company). Translation: "Divination in the day of Gengchen: Shall we pray to (some god) for ending the locust plague? ……". In addition, the "locust" characters, highlighted by red boxes, are shown beside the rubbings. In North China, droughts in summer always cause the highest regional locust outbreaks (*107*). (**B**) Variations in "pray for rain" pieces ratio (proportions of oracle bone script pieces that contain inquiries about "pray for rain" in the five oracle bone script phases, Fig. S6C) and "locust-like character" pieces ratio (proportions of oracle bone script pieces that contain locust-like characters in the five oracle bone script phases, Fig. S6D) (*108*), and El Niño-caused sediments (the solid grey line denotes the 100-point running average) (*68*). The different widths of the red and yellow bars represent the time range of each oracle bone script phase. (**C**) Comparison of the pray for rain record ratio and the groundwater level changes in Late Shang. The width of each arrow (tail) shows a time to which the age of certain groundwater level data belongs (*109*). (**D**) Rainfall anomaly ratio in strong El Niño developing years (1982, 1997, 2015) compared to the average (1950–2021). The vectors give the wind field anomaly at 850 hPa.

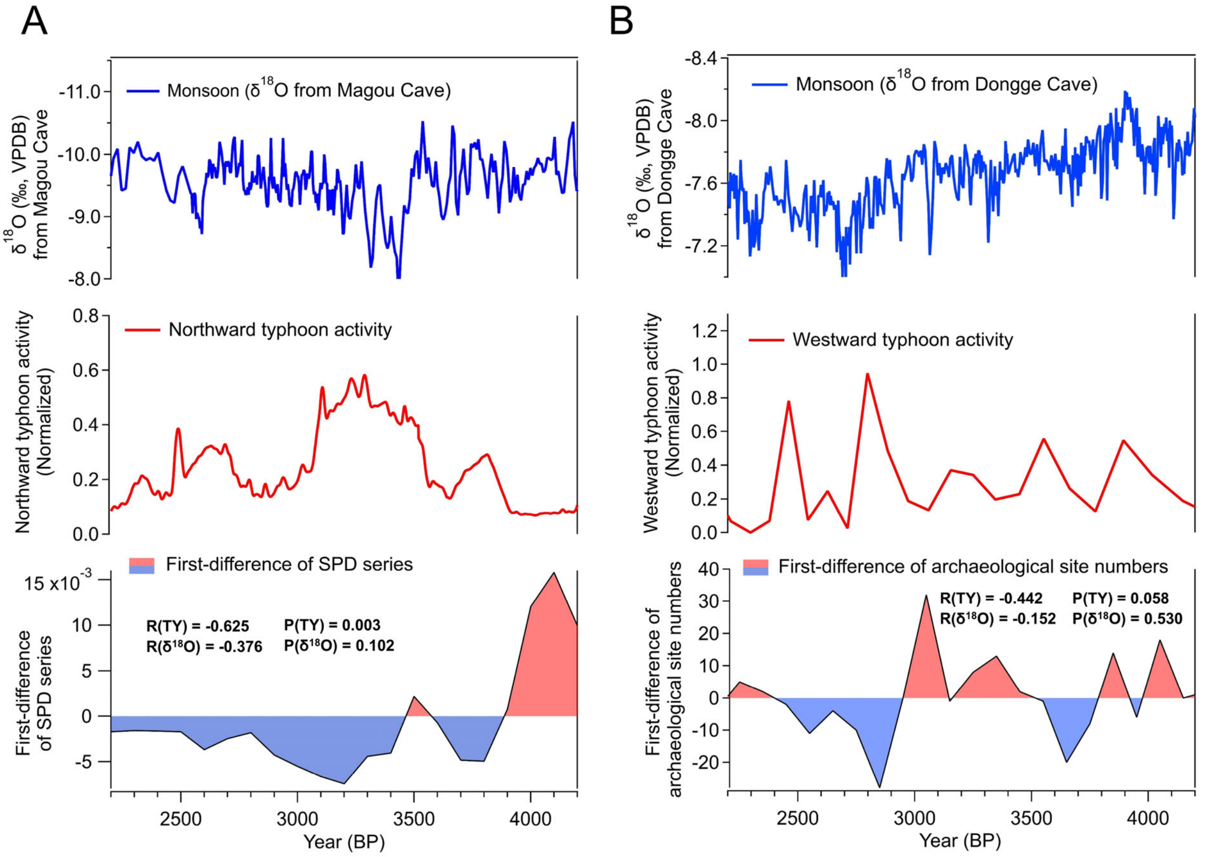

Fig. S17. Monsoon, typhoon, and population dynamics around 3000 BP in the Central Plains and Chengdu Plain. (**A**) Central Plains: monsoon intensity (composite stalagmite $\delta^{18}O$ time series from Magou Cave (*110*)), northward typhoon activity (this study), and population change (first-difference of SPD series, later minus previous). (**B**) Chengdu Plain: monsoon intensity ($\delta^{18}O$ time series from Dongge Cave (*111*)), westward typhoon activity (*26*), and population change (first-difference of archaeological site numbers, later minus previous). Magou and Dongge caves are proximal to the Central Plains and Chengdu Plain, respectively. Notation: R(X) = Pearson correlation between population change and target series X; P(X) = p-value for R(X). Correlations are computed after linear detrending of both series. Abbreviations: $\delta^{18}O$ = cave-calcite oxygen-isotope record (Magou in (A); Dongge in (B)); TY = typhoon activity (northward in (A); westward in (B)).

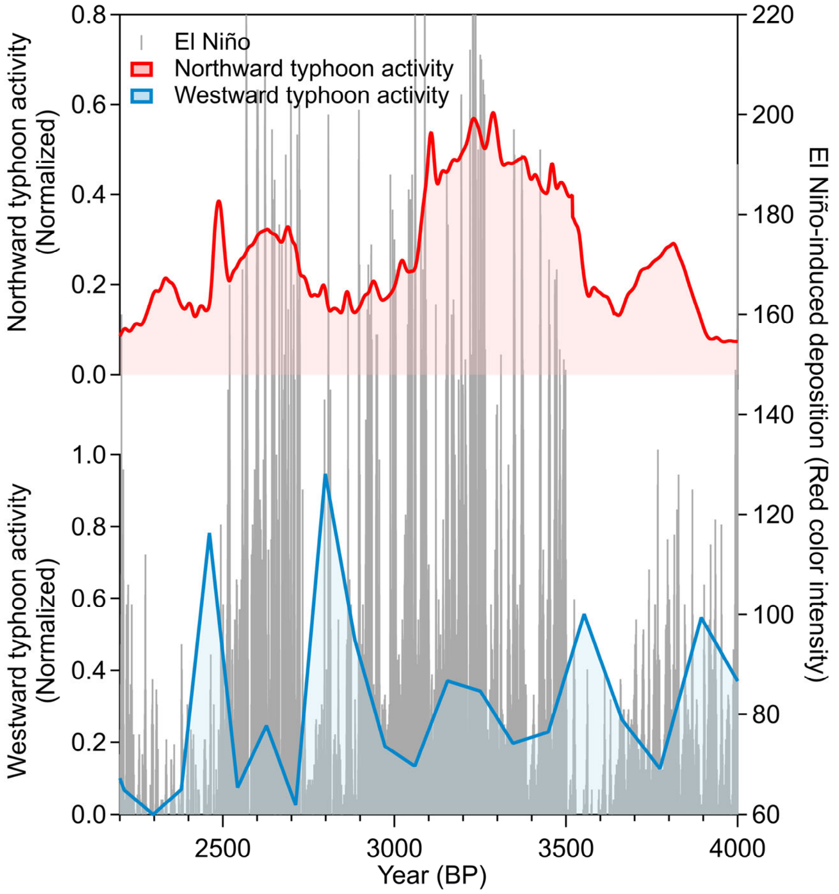

Fig. S18. Relationship between typhoon and El Niño activity. The northward (westward) typhoon activities were normalized from the typhoon proxies got in YR, NR and KI (PR) (*23–26*). The grey lines indicate the variations in El Niño activities reconstructed by Moy et al. (2002) (*68*).

Table S1. Archaeological sites of the Ancient Shu civilization in the Chengdu Plain

Site No.	Archaeological site		Location	
1	Guiyuanqiao site		104.220 °E	31.110 °N
2	Baodun site		103.750 °E	30.433 °N
3	Pixian city site		103.917 °E	30.900 °N
4	Yufucun site		103.840 °E	30.760 °N
5	Mangcheng site		103.583 °E	30.867 °N
6	Shuanghe city site		103.600 °E	30.767 °N
7	Gewei Pharmaceutical site	“Gewei Pharmaceutical Phase 1” locality	103.947 °E	30.743 °N
8		Mofu Biological Technology locality	103.944 °E	30.744 °N
9		Huili Packaging Factory locality	103.945 °E	30.743 °N
10	Caojiaci site		103.887 °E	30.781 °N
11	Sanxingdui site		104.210 °E	31.000 °N
12	Hangkonggang site		103.950 °E	30.770 °N
13	Sanxingcun site		104.220 °E	30.890 °N
14	Guilinxiang site		104.180 °E	30.820 °N
15	Qingjiangcun site		103.817 °E	30.750 °N
16	“Wanan Pharmaceutical Packaging Factory” site		103.953 °E	30.753 °N
17	“Microwave Production Base” site		103.954 °E	30.753 °N
18	Yongfucun Group III site		103.896 °E	30.722 °N
19	Chenjiayuanzi site		103.815 °E	30.824 °N
20	Fanjianian site		103.892 °E	30.694 °N
21	Hongfengcun site		104.220 °E	30.890 °N
22	Songjia Heba site		103.940 °E	30.870 °N
23	Sanguancun site		103.941 °E	30.791 °N
24	Zhongyi site		104.065 °E	30.820 °N
25	Zhujiacun site		104.222 °E	30.833 °N
26	New Compus of Southwest Jiaotong University site		103.990 °E	30.770 °N
27	Fangyuan Zhongke site		103.959 °E	30.752 °N
28	Qinggangcun site		103.806 °E	30.807 °N
29	Minjiang Xiaoqu site		104.080 °E	30.620 °N
30	Liuhecun site		103.883 °E	30.483 °N
31	Tianxianglu site		103.829 °E	30.728 °N
32	Yanduizi site		104.280 °E	31.100 °N
33	Zhengyin site		104.200 °E	30.830 °N

34	Xinyicun site		104.050 °E	30.670 °N
35	Dajiangcun site		104.070 °E	30.810 °N
36	Xiquguoji site		103.954 °E	30.733 °N
37	Zhonghai Guoji site	Zhonghai Guoji, locality No. 1	103.970 °E	30.727 °N
38		Zhonghai Guoji, locality No. 2	103.974 °E	30.720 °N
39		Zhonghai Guoji, locality No. 3	103.974 °E	30.720 °N
40		Zhonghai Guoji, locality No. 4	103.973 °E	30.727 °N
41	Jinsha site	South Furongyuan locality	104.020 °E	30.690 °N
42		Sanhehuayuan locality	104.020 °E	30.700 °N
43		Gandao Huangzhong line-A locality	104.011 °E	30.687 °N
44		Gandao Huangzhong line-B locality	104.011 °E	30.687 °N
45		Wanbo locality	104.010 °E	30.690 °N
46		Lanyuan locality	104.500 °E	30.684 °N
47		Zongzhuang Logistics Supply Station locality	104.030 °E	30.690 °N
48		Renfang locality	104.500 °E	30.684 °N
49		Guoji Huayuan locality	104.700 °E	30.691 °N
50		Shufeng Huayuan Phase 2 locality	104.500 °E	30.684 °N
51		Chunyu Huajian locality	104.010 °E	30.700 °N
52		Jingpinfang locality	104.010 °E	30.710 °N
53		Qiangyi Automobile Trading Company locality	104.013 °E	30.723 °N
54		Westward Extension of Xinghe Road locality	104.900 °E	30.695 °N
55		Xicheng Tianxia locality	104.020 °E	30.700 °N
56		Ruyang locality	103.983 °E	30.708 °N
57		"Zhixin Jinsha Phase 1" locality	104.800 °E	30.679 °N
58		Longzui Extension line-B locality	104.010 °E	30.690 °N
59		Huanghe locality	104.700 °E	30.686 °N
60		Huangzhong Elementary School locality	104.013 °E	30.689 °N
61	Shi'erqiao site		104.050 °E	30.670 °N
62	Taipingcun site		104.012 °E	30.861 °N
63	Futong Optical Cable site		103.944 °E	30.762 °N
64	Zhuwangcun site		104.208 °E	30.842 °N

65	Xinzhuangcun site	103.910 °E	30.720 °N
66	Hongqiaocun site	103.890 °E	30.692 °N
67	Laboratory Building (University of Electronic Science and Technology of China) site	103.928 °E	30.756 °N
68	Administrative Building (University of Electronic Science and Technology of China) site	103.928 °E	30.756 °N
69	Shijiefang site	104.080 °E	30.620 °N
70	Huachengcun site	104.040 °E	30.690 °N
71	“Shunjiang Xiaoqu Phase 3” site	103.921 °E	30.743 °N
72	“Shunjiang Xiaoqu Phase 2” site	103.921 °E	30.743 °N
73	Gaoqiaocun site	104.210 °E	30.830 °N
74	Tuanjiecun site	104.151 °E	30.813 °N
75	“Jinhai'an Phase 2” site	104.420 °E	30.860 °N
76	Liu'ancun site	103.778 °E	30.754 °N
77	Xinhuacun site	104.294 °E	30.844 °N
78	Hetaocun site	104.030 °E	30.630 °N
79	Shuzhuangtai site	103.734 °E	30.900 °N
80	Lijia Yuanzi site	103.843 °E	30.853 °N
81	“Languang Organic Beverage Phase 2” site	103.891 °E	30.780 °N
82	Meihuaquan site	103.981 °E	30.945 °N
83	Mishaiquan site	103.981 °E	30.945 °N
84	Tiantaicun site	103.910 °E	30.808 °N
85	“Guoteng Phase 2” site	103.966 °E	30.730 °N
86	"Putian Cable" site	103.954 °E	30.733 °N
87	Tianwangcun site	103.910 °E	30.733 °N
88	Qinglongcun site	104.100 °E	30.940 °N
89	Dafucun site	104.280 °E	30.870 °N
90	“Datang Telecom Phase 2” site	103.966 °E	30.741 °N
91	Fuqin Xiaoqu site	104.040 °E	30.690 °N
92	Fangchijie site	104.060 °E	30.660 °N
93	Longquancun site	103.920 °E	30.950 °N
94	Xia Dongdajie site	104.090 °E	30.650 °N
95	New Location of Chengdu Museum site	104.070 °E	30.660 °N
96	Qingyanggong site	104.050 °E	30.670 °N
97	Shang Wangjiaguai site	104.060 °E	30.660 °N
98	Shuiguanyin site	104.020 °E	30.880 °N
99	Zhihuijie site	104.067 °E	30.667 °N
100	Xinjinxi Packaging Factory site	103.959 °E	30.751 °N

101	Zhaojia Heba site		104.064 °E	30.925 °N
102	Xihua University site	School of Computer and Software Engineering locality	103.954 °E	30.785 °N
103		The 6th Teaching Building locality	103.954 °E	30.781 °N
104		Art School locality	103.954 °E	30.785 °N
105	North Extension of Tianfu Avenue (Deyang Section) sites	Yongle site	104.277 °E	31.052 °N
106		Lianhe site	104.249 °E	31.051 °N
107		Gaoshiqiao site	104.246 °E	31.012 °N
108		Lanjia site	104.244 °E	31.800 °N
109		Tuanbai site	104.180 °E	30.921 °N
110	Duck River Basin sites	Jiangjunmiao site	104.213 °E	31.027 °N
111		Zhengjianian site	104.173 °E	31.039 °N
112		Paqiang Tudi site	104.145 °E	31.063 °N
113		Liujia Yuanzi site	104.110 °E	31.083 °N
114		Taojia Aiqiang site	104.106 °E	31.087 °N
115		Zhujia Citang site	104.074 °E	31.123 °N
116		Baimiaozi site	104.141 °E	31.044 °N
117	Xinghuocun site		103.858 °E	30.882 °N
118	Gaoshan city site		103.580 °E	30.453 °N
119	Yandian city site		103.514 °E	30.553 °N
120	Zizhu city site		103.594 °E	30.565 °N
121	Jiantaicun site		104.168 °E	31.112 °N
122	Zhoujia Yuanzi site		103.988 °E	30.968 °N
123	Fengjia Yuanzi site		103.670 °E	30.956 °N
124	Xinyaopu site		104.186 °E	31.029 °N
125	Tongmengcun site		103.978 °E	30.902 °N

References of the archaeological data of these sites are listed in Data S1.

Table S2. Representative Modern Typhoon-Related Flood Disasters in Inland China

Time	Typhoon	Flooding region	Impacts
1975/08	Nina	Southern Henan Province	Affected 29 counties, 11 million people, and over 11,000 km^2 of farmland; 7,300 km^2 severely damaged. Flooded grain: ~2 billion kg. ~102 km of the Beijing–Guangzhou Railway were destroyed. Estimated deaths: ~260,000.
2021/07	In-Fa	Henan Province	Affected 14.786 million people across 150 counties in 16 cities; 398 people were reported dead or missing; 1.49 million were urgently relocated; 39,000 houses collapsed, 171,000 severely damaged, and 616,000 moderately damaged; 873,500 hectares of crops were affected; direct economic losses reached 120.06 billion RMB.
2023/07	Doksuri	Beijing-Tianjin-Hebei	Affected 5.51 million people in Beijing-Tianjin-Hebei; 107 deaths or missing; 1.43 million relocated; over 1.3 million houses damaged or destroyed; 416,100 hectares of crops affected; direct economic losses: 165.79 billion RMB.
1995/08	Helen	Chengdu and surrounding areas	The "95.8" flood event; crops affected over 427,000 hectares, including 38,000 hectares of total loss; 8,000 hectares of farmland washed away. Grain production reduced by 1.21 billion kg, with 5.6 million kg of stored grain lost. Over 720 km of roadbeds were destroyed and 426 bridges and culverts were damaged. 310,000 people were trapped by floods; direct economic losses reached 2.45 billion RMB.

References including:
1975/08 case: Hu & Luo, 1989 (*48*)
2021/07 case: https://www.mem.gov.cn/xw/yjglbgzdt/202201/t20220123_407199.shtml
2023/07 case: https://www.mem.gov.cn/xw/yjglbgzdt/202401/t20240120_475696.shtml
1995/08 case: Zhan, 2006 (*46*)

Table S3. Phases of oracle bone scripts and the time span

Phase number	Phases (shown as name(s) of the king(s))	Time span
1	Wuding (武丁 in Chinese)	3200–3142 BP
2	Zugeng, Zujia (祖庚, 祖甲 in Chinese)	3141–3098 BP
3	Linxin, Kangding (廪辛, 康丁 in Chinese)	
4	Wuyi, Wending (武乙, 文丁 in Chinese)	3097–3052 BP
5	Diyi, Dixin (帝乙, 帝辛 in Chinese)	3051–2996 BP

According to the age-dating of the five phases of oracle bone scripts, phases 2 to 3 are around 3141–3098 BP. We roughly divide this timespan into two equal parts.

Table S4. Oracle bone scripts data source

**Oracle bone script data source (translation)**	**Oracle bone script data source in Chinese**
Guo, M. (Ed.) (1978–1983). *The Complete Collection of Oracle Bone Inscriptions (1–13)*. Beijing: Zhonghua Book Company.	郭沫若主编，胡厚宣总编辑，中国社会科学院历史研究所编辑：《甲骨文合集》（1–13），北京：中华书局，1978–1983 年。
Hu, H. (Ed.) (1999). *Interpretation of the Complete Collection of Oracle Bone Inscriptions (1–4)*. Beijing: China Social Science Press.	胡厚宣主编：《甲骨文合集释文》（1–4），北京：中国社会科学出版社，2009 年。
Institute of Archaeology CASS (Ed.) (1980). *The Oracle Bones at South Xiaotun Site, Volume One (1–2)*. Beijing: Zhonghua Book Company.	中国社会科学院考古研究所编著：《小屯南地甲骨（上册）》（1–2），北京：中华书局，1980 年。
Institute of Archaeology CASS (Ed.) (1984). *The Oracle Bones at South Xiaotun Site, Volume Two (1–3)*. Beijing: Zhonghua Book Company.	中国社会科学院考古研究所编著：《小屯南地甲骨（下册）》（1–3），北京：中华书局，1984 年。
Yao, X., & Xiao, D. (Eds.). (1989). *Collection and Classification of Inscriptions on Bones and Tortoise Shells in Yinxu*. Beijing: Zhonghua Book Company.	姚孝遂主编、肖丁副主编：《小屯南地甲骨考释》，北京：中华书局，1985 年。
Li, X., Qi, W. & Ai, L. (Eds.). (1985). *Oracle Bone Collections in Great Britain, Volume One (1–2)*. Beijing: Zhonghua Book Company.	李学勤、齐文心、艾兰编著：《英国所藏甲骨集（上编）》（上、下），北京：中华书局，1985 年。
Li, X., Qi, W. & Ai, L. (Eds.). (1985). *Oracle Bone Collections in Great Britain, Volume Two (1–2)*. Beijing: Zhonghua Book Company.	李学勤、齐文心、艾兰编著：《英国所藏甲骨集（下编）》（上、下），北京：中华书局，1992 年。
Peng, B., Xie, J. & Ma, J. (Eds.). (1999). *Supplement to Oracle Bone Script Complications (1–7)*. Beijing: Language & Culture Press.	彭邦炯、谢济、马季凡编著：《甲骨文合集补编》（1–7），北京：语文出版社，1999 年。
Institute of Archaeology CASS (Ed.) (2003). *The Oracle Bones at East Huanyuanzhuang Site in Yinxu (1–6).* Kunming: Yunnan People's Publishing House.	中国社会科学院考古研究所编著：《殷墟花园庄东地甲骨》（1–6），昆明：云南人民出版社，2003 年。

Table S5. Quantitative analyses of oracle bone scripts in the five phases

Phase number	Total count (pieces)	Containing "rainfall" (count, proportion)	Containing "heavy rainfall" (count, proportion)
1	29061	2450 (8.43%)	55 (1.89‰)
2	6616	423 (6.39%)	8 (1.21 ‰)
3	7855	1175 (14.96%)	132 (16.68‰)
4	5341	709 (13.27%)	22 (4.12‰)
5	6600	126 (1.91%)	2 (0.30‰)

Table S6. Flood deposits during periods of intensified typhoon activities around 3000 BP

Flood deposits site		Location	Time of flood deposits	Reference
North China Plain	Neiqiu profile	114.52 °E, 34.29 °N	13000±140BP<br>12910±180 BP<br>10530±120 BP<br>9500±40 BP<br>8760±250 BP<br>5600±85 BP<br>5020±200 BP<br>4600±190 BP<br>**3470±125 BP**	C. Yin, W. Qiu, R. Li, Holocene Paleofloods in the North China Plain. *Journal of Beijing Normal University (Natural Science)* **37**, 280–284 (2001). (in Chinese) (*112*)
	**Suning profile**	115.85 °E, 38.43 °N		
Yihe River Basin	**Beizhai profile**	118.43 °E, 35.55 °N	4100–3800 BP<br>**3300–3000 BP**<br>900–200 BP	H. Shen *et al.*, OSL and radiocarbon dating of flood deposits and its paleoclimatic and archaeological implications in the Yihe River Basin, East China. *Quaternary Geochronology* **30**, 398–404 (2015). (*113*)
Jiaozuo city	**Jiayingguan profile**	113.45 °E, 34.94 °N	**2900–2500 BP**<br>1200–800 BP	J. Shi, *Study on Sedimentary Environment of Holocene Slackwater Deposits in the Jiaozuo Section of the Yellow River*, Jiangsu Normal University, Xuzhou (2019). (*114*)
Heze city	**Shilipu sites**	115.53 °E, 35.12 °N	**3.78±0.36 ka (OSL age)**<br>**3.59±0.25 ka (OSL age)**<br>**3.56±0.39 ka (OSL age)**<br>**2.62±0.24 ka (OSL age)**<br>0.86±0.07 ka (OSL age)	S.-Y. Yu *et al.*, Extreme flooding of the lower Yellow River near the Northgrippian-Meghalayan boundary: Evidence from the Shilipu archaeological site in southwestern Shandong Province, China. *Geomorphology* **350**, 106878 (2020). (*115*)
Middle Yiluohe River valley	**Longmenxia profile**	112.49 °E, 34.54 °N	**3100–3000 BP**<br>1800–1700 BP<br>770–610 BP<br>420–340 BP	X. Zhao *et al.*, Holocene climatic events recorded in palaeoflood slackwater deposits along the middle Yiluohe River valley, middle Yellow River basin, China. *Journal of Asian Earth Sciences* **123**, 85–94 (2016). (*116*)
Anyang city	**Sanyangzhuang profile**	35.73 °N, 114.77 °E	**3.91±0.27 ka (OSL age)**<br>**3.15±0.20 ka (OSL age)**<br>**3.14±0.22 ka (OSL age)**<br>**2.97±0.19 ka (OSL age)**<br>**2.59±0.17 ka (OSL age)**	M. Yang *et al.*, Optically stimulated luminescence dating of Sanyangzhuang profile,Henan Province. *Journal of Earth Environment* **9**, 580–588 (2019). (in Chinese). (*117*)

Table S7. Model configuration options and settings

**Domain setting**	**1976 case**	**1995 case**
Horizontal grid	280 × 300	280 × 300
Grid spacing	20 km × 20 km	20 km × 20 km
Vertical layers	41 eta levels	41 eta levels
Centre point	120 °E, 30 °N	120 °E, 30 °N
Map projection	Lambert	Lambert
**Paramerization configuration**		
Long-wave radiation	Dudhia scheme	Dudhia scheme
Short-wave radiation	Dudhia scheme	Dudhia scheme
Cumulus parameterization	Kain-Fritsch	Kain-Fritsch
Land-surface	Noah	Noah
PBL	YSU	YSU
Microphysics	WRF Single-Moment 3-class scheme	WRF Single-Moment 3-class scheme

Table S8. Count of oracle bone pieces with typical verification statement in the five phases

Phase number	Total count (pieces) containing "rainfall" and "pray for rainfall"	Total count (pieces) containing "rainfall" and "pray for rainfall" with typical verification statement
1	3448	110
2	424	16
3	1230	2
4	780	7
5	126	1

Typical verification statement here refers to oracle bone scripts with the character "允" along with inquiry of rainfall (*108*), which means the rainfall is "as expected" or "not as expected" in the verification statement.

Data S1 (separate file)

Data S1. References of the archaeological data for sites in the Chengdu Plain

REFERENCES

1. S. I. Seneviratne, X. Zhang, M. Adnan, W. Badi, C. Dereczynski, A. Di Luca, S. Ghosh, I. Iskandar, J. Kossin, S. Lewis, F. Otto, I. Pinto, M. Satoh, S.M. Vicente-Serrano, M. Wehner, B. Zhou, "Weather and climate extreme events in a changing climate," in *Climate Change 2021: The Physical Science Basis. Contribution of Working Group I to the Sixth Assessment Report of the Intergovernmental Panel on Climate Change*, V. Masson-Delmotte, P. Zhai, A. Pirani, S. L. Connors, C. Péan, S. Berger, N. Caud, Y. Chen, L. Goldfarb, M. I. Gomis, M. Huang, K. Leitzell, E. Lonnoy, J. B. R. Matthews, T. K. Maycock, T. Waterfield, O. Yelekçi, R. Yu, B. Zhou, Eds. (Cambridge Univ. Press, 2023), pp. 1513–1766.

2. R. Grove, G. Adamson, "El Niño in prehistory," in *El Niño in World History* (Palgrave Macmillan, 2018), pp. 19–48.

3. T. H. Donders, F. Wagner, D. L. Dilcher, H. Visscher, Mid- to late-Holocene El Niño-Southern Oscillation dynamics reflected in the subtropical terrestrial realm. *Proc. Natl. Acad. Sci. U.S.A.* **102**, 10904–10908 (2005).

4. H. Zhang, H. Cheng, A. Sinha, C. Spötl, Y. Cai, B. Liu, G. Kathayat, H. Li, Y. Tian, Y. Li, J. Zhao, L. Sha, J. Lu, B. Meng, X. Niu, X. Dong, Z. Liang, B. Zong, Y. Ning, J. Lan, R. L. Edwards, Collapse of the Liangzhu and other Neolithic cultures in the lower Yangtze region in response to climate change. *Sci. Adv.* **7**, eabi9275 (2021).

5. R. A. Bryson, H. H. Lamb, D. L. Donley, Drought and the decline of Mycenae. *Antiquity* **48**, 46–50 (1974).

6. S. W. Manning, C. Kocik, B. Lorentzen, J. P. Sparks, Severe multi-year drought coincident with Hittite collapse around 1198–1196 BC. *Nature* **614**, 719–724 (2023).

7. L. Liu, X. Chen, H. Wright, H. Xu, Y. Li, G. Chen, H. Zhao, H. Kim, G.-A. Lee, Rise and fall of complex societies in the Yiluo region, North China: The spatial and temporal changes. *Quat. Int.* **521**, 4–15 (2019).

8. W. Gao, H. Yuan, Y. Pan, W. Jia, X. Liu, K. Li, Spatiotemporal variation of human settlement distribution between the Shang and Western Zhou dynasties in relation to flooding in the lower Yellow River floodplain, East China. *J. Archaeol. Sci. Rep.* **52**, 104260 (2023).

9. C. Zhu, J. Xu, T. Jia, M. Zeng, M. Huang, *Environmental Archaeology of the Rise and Fall of Sanxingdui and Jinsha Civilizations in Sichuan* (Nanjing Univ. Press, Nanjing, 2021) (in Chinese).

10. C. Zhu, J. Xu, M. Huang, Z. Yang, N. Zhang, Z. Jiang, T. Bai, F. Lu, Archaeological discoveries and research on the remains of an ancient flood event at the Majie Site in the Chengdu Plain. *Earth Science Frontiers* **28**, 181–201 (2021).

11. F. Chen, Q. Xu, J. Chen, H. J. B. Birks, J. Liu, S. Zhang, L. Jin, C. An, R. J. Telford, X. Cao, Z. Wang, X. Zhang, K. Selvaraj, H. Lu, Y. Li, Z. Zheng, H. Wang, A. Zhou, G. Dong, J. Zhang, X. Huang, J. Bloemendal, Z. Rao, East Asian summer monsoon precipitation variability since the last deglaciation. *Sci. Rep.* **5**, 11186 (2015).

12. F. Lu, J. Dodson, W. Zhang, H. Yan, Mid to late Holocene environmental change and human impact: A view from Central China. *Quat. Sci. Rev.* **223**, 105953 (2019).

13. J. Wu, C. M. Shen, H. Yang, S. Qian, S. C. Xie, Holocene temperature variability in China. *Quat. Sci. Rev.* **312**, 108184 (2023).

14. Xia Shang Zhou Chronology Project Team, Ed. "Chronological research on the Late Shang period," in *Research Report of Xia Shang Zhou Chronology Project* (Science Press, 2022), pp. 186–249 (in Chinese).

15. R. L. Thorp, *China in the Early Bronze Age: Shang Civilization* (University of Pennsylvania Press, 2006).

16. K. Bi, L. Xie, H. Zhang, X. Chen, X. Gu, Q. Tian, Accurate medium-range global weather forecasting with 3D neural networks. *Nature* **619**, 533–538 (2023).

17. L. Liu, X. Chen, *The Archaeology of China: From the Late Paleolithic to the Early Bronze Age* (Cambridge Univ. Press, 2012).

18. C. Shen, *Anyang and Sanxingdui: Unveiling the Mysteries of Ancient Chinese Civilizations* (Royal Ontario Museum, 2002).

19. Y. Kang, X. Peng, S. Wang, Y. Hu, K. Shang, S. Lu, Observational analyses of topographic effects on convective systems in an extreme rainfall event in Northern China. *Atmos. Res.* **229**, 127–144 (2019).

20. Y. Chen, Y. Li, Convective characteristics and formation conditions in an extreme rainstorm on the eastern edge of the Tibetan Plateau. *Atmos.* **12**, 381 (2021).

21. R. Zhao, B. Chen, W. Zhang, S. Yang, X. Xu, Formation mechanisms of persistent extreme precipitation events over the eastern periphery of the Tibetan Plateau: Synoptic conditions, moisture transport and the effect of steep terrain. *Atmos. Res.* **304**, 107341 (2024).

22. C. Wang, H. Lu, J. Zhang, Z. Gu, K. He, Prehistoric demographic fluctuations in China inferred from radiocarbon data and their linkage with climate change over the past 50,000 years. *Quat. Sci. Rev.* **98**, 45–59 (2014).

23. J. D. Woodruff, J. P. Donnelly, A. Okusu, Exploring typhoon variability over the mid-to-late Holocene: Evidence of extreme coastal flooding from Kamikoshiki, Japan. *Quat. Sci. Rev.* **28**, 1774–1785 (2009).

24. J. Lim, J.-Y. Lee, S.-S. Hong, J.-Y. Kim, Late Holocene flooding records from the floodplain deposits of the Yugu River, South Korea. *Geomorphology* **180-181**, 109–119 (2013).

25. J. Lim, J.-Y. Lee, S.-S. Hong, J.-Y. Kim, S. Yi, W.-H. Nahm, Holocene changes in flooding frequency in South Korea and their linkage to centennial-to-millennial-scale El Nino-Southern Oscillation activity. *Quatern. Res.* **87**, 37–48 (2017).

26. P. Li, M. Li, H. Gan, Z. Xia, A preliminary study on sediment records of possible typhoon in the northern South China Sea during the past 6500 yr. *Holocene* **31**, 1221–1228 (2021).

27. C. Zhang, Longshan-Erlitou cultures—Changing cultural patterns in prehistoric China and the emergence of the globalization in the Bronze Age. *Cultural Relics* **6**, 50–59 (2017).

28. Y. Feng, “The decline and use of the early Bronze Age capitals in the Zhengluo region,” thesis, Shandong University, Ji’nan, China (2023) (in Chinese).

29. W. Hou, *Study on Settlement Archaeology in Zhengzhou Shang City and the Vicinity of Capital* (China Social Sciences Press, 2024) (in Chinese).

30. Y. Liu, D. Zhang, Archaeological observation of social restructuring during Late Shang in modern-day Zhengzhou. *Huaxia Archaeology* **2**, 62–68 (2024).

31. Institute of Archaeology, Chinese Academy of Social Sciences (CASS), Ed. in *Chinese Archaeology: Western Zhou and Eastern Zhou* (China Social Sciences Press, 2004) (in Chinese).

32. T. Pang, Ed. in *Encyclopedia of Meteorological Disasters in China* (China Meteorological Press, 2005), vol. Henan (in Chinese).

33. L. Yang, Y. Yang, J. Smith, The upper tail of flood peaks over China: Hydrology, hydrometeorology, and hydroclimatology. *Water Resour. Res.* **57**, e2021WR030883 (2021).

34. Y. Ding, On the study of the unprecedented heavy rainfall in Henan Province during 4-8 August 1975: Review and assessment. *Acta. Meteor. Sin.* **73**, 411–424 (2015).

35. Y. Nie, J. Sun, Moisture sources and transport for extreme precipitation over Henan in July 2021. *Geophys. Res. Lett.* **49**, e2021GL097446 (2022).

36. K.-c. Chang, *Shang Civilization* (Yale Univ. Press, 1980).

37. D. N. Keightley, *Sources of Shang History: The oracle-bone inscriptions of Bronze Age China* (University of California Press, 1985).

38. K. A. Wittfogel, Meteorological records from the divination inscriptions of Shang. *Geogr. Rev.* **30**, 110–133 (1940).

39. Z. Yue, H. Yue, □□□□□□□□□□□. *Yindu J.* **1**, 35–38 (2012) [An inquiry into the causes of the desruction of the Yin ruins capital].

40. Y. He, Huanbei Shang City and Yinxu waterway systems and related issues. *Archaeology* **9**, 82–94 (2021).

41. X. Zhang, “Water conservancy of Shang dynasty urban settlements,” in *Study on the Water Conservancy of the Shang Dynasty* (China Social Science Press, 2015), pp. 179–264 (in Chinese).

42. X. Yu, Ed., “Disaster,” in *Textual Research and Explanation of Inscriptions on Oracle Bones* (Zhonghua Book Company, 1996), pp. 1292–1296 (in Chinese).

43. Y. Li, S. Zhao, Floods losses and hazards in China from 2001 to 2020. *Clim. Chang. Res.* **18**, 154–165 (2022).

44. S. Zhao, G. Gao, D. Huang, W. He, Characteristics of meteorological disaster losses in China from 2004 to 2013. *J. Meteorol. Environ.* **33**, 101–107 (2017).

45. W. Feng, L. Cheng, M. Cheng, Nonhydrostatic numerical simulation for the “96.8” extraordinary rainstorm and the developing structure of mesoscale system. *Acta. Meteor. Sin.* **59**, 294–307 (2001).

46. Z. Zhan, Ed., “Heavy rain and floods,” in *Encyclopedia of Meteorological Disasters in China* (China Meteorological Press, 2006), vol. Sichuan, pp. 189–190 (in Chinese).

47. Q. Gu, L. Kang, Y. Zhang, Analysis of a heavy torrential rainfall in west Sichuan in August of 1995. *J. Chengdu Inst. Meteorol.* **41**, 177–184 (1997).

48. M. Hu, C. Luo, Eds. in *Major Floods in Chinese History (Vol. 2)* (China Bookstore, 1989) (in Chinese).

49. G. Hu, C. C. Huang, Y. Zhou, J. Pang, X. Zha, Y. Guo, Y. Zhang, X. Zhao, Hydrological studies of the historical and palaeoflood events on the middle Yihe River, China. *Geomorphology* **274**, 152–161 (2016).

50. X. Liu, □□□□□□□□□□. *Archaeology* **1**, 33–39 (1961) [Excavation of the Jianxi site in Mengxian County, Henan Province].

51. Sichuan Provincial Institute of Cultural Relics and Archaeology, Chengdu Municipal Institute of Cultural Relics and Archaeology, Eds. in *Shi'erqiao site in Chengdu* (Cultural Relics Press, 2009) (in Chinese).

52. J. Wan, □□□□□□□□□□□□□□□□□□□. *Cultural Relics* **12**, 38–47 (2017) [An analysis of the nature and formation of mechanisms of early deposits at the Shi'qiao site in Chengdu].

53. P. Xu, Excavation of the Fangchijie site in Chengdu city. *Acta Archaeologica Sinica* **2**, 297–316 (2003).

54. Z. Jiang, The analysis of evolution of the Pre-Qin settlements in Chengdu Plain. *Archaeology* **4**, 67–78 (2015).

55. J. Zheng, W.-C. Wang, Q. Ge, Z. Man, P. Zhang, Precipitation variability and extreme events in eastern China during the past 1500 years. *Terr. Atmos. Ocean. Sci.* **17**, 579–592 (2006).

56. Changjiang Water Resources Commission of the Ministry of Water Resources, Chongqing Municipal Bureau of Culture, Chongqing Museum, Eds. in *Collected Historical Materials on Flooding in Sichuan across Two Millennia* (Cultural Relics Press, 1993) (in Chinese).

57. K. Wen, Ed. in *Encyclopedia of Meteorological Disasters in China* (China Meteorological Press, 2008) (in Chinese).

58. D. Zhang, Ed. in *A Compendium of Chinese Meteorological Records of the Last 3,000 Years* (Jiangsu Education Press, ed. 2, 2013) (in Chinese).

59. M. Carré, P. Braconnot, M. Elliot, R. d'Agostino, A. Schurer, X. Shi, O. Marti, G. Lohmann, J. Jungclaus, R. Cheddadi, I. A. di Carlo, J. Cardich, D. Ochoa, R. Salas Gismondi, A. Pérez, P. E. Romero, B. Turcq, T. Corrège, S. P. Harrison, High-resolution marine data and transient simulations support orbital forcing of ENSO amplitude since the mid-Holocene. *Quat. Sci. Rev.* **268**, 107125 (2021).

60. R. Xing, Z. Ding, S. You, H. Xu, Relationship of tropical-cyclone-induced remote precipitation with tropical cyclones and the subtropical high. *Front. Earth Sci.* **10**, 595–606 (2016).

61. L. Chen, Y. Xu, Review of typhoon very heavy rainfall in China. *Meteor. Environ. Sci.* **40**, 3–10 (2017).

62. J. Tang, X. Xu, W. Cai, C. Wang, Water vapour multi-vortex structure under the interactions of typhoons and mid-low latitude systems during extreme precipitation in North China. *Adv. Clim. Chang. Res.* **14**, 116–125 (2023).

63. Z. Chen, F. Huang, G. He, A case study of interactions between the tropical cyclone and the southwest vortex. Part I: Diagnostic analysis. *Chin. J. Atmos. Sci.* **26**, 352–360 (2002).

64. G. Zhou, T. Shen, Y. Han, A numerical simulation and diagnoses case analysis of typhoon affect on southwest vortex. *Sci. Meteor. Sinica* **26**, 620–626 (2006).

65. C. M. Brandon, J. D. Woodruff, D. P. Lane, J. P. Donnelly, Tropical cyclone wind speed constraints from resultant storm surge deposition: A 2500 year reconstruction of hurricane activity from St. Marks, FL. *Geochem. Geophys. Geosyst.* **14**, 2993–3008 (2013).

66. J. D. Woodruff, K. Kanamaru, S. Kundu, T. L. Cook, Depositional evidence for the Kamikaze typhoons and links to changes in typhoon climatology. *Geology* **43**, 91–94 (2015).

67. P. C. Banacos, D. M. Schultz, The use of moisture flux convergence in forecasting convective initiation: Historical and operational perspectives. *Weather Forecast* **20**, 351–366 (2005).

68. C. M. Moy, G. O. Seltzer, D. T. Rodbell, D. M. Anderson, Variability of El Nino/Southern Oscillation activity at millennial timescales during the Holocene epoch. *Nature* **420**, 162–165 (2002).

69. N. Wen, Z. Liu, Y. Liu, Direct impact of El Nino on East Asian summer precipitation in the observation. *Climate Dynam.* **44**, 2979–2987 (2015).

70. A. B. Frappier, J. Pyburn, A. D. Pinkey-Drobnis, X. Wang, D. R. Corbett, B. H. Dahlin, Two millennia of tropical cyclone-induced mud layers in a northern Yucatán stalagmite: Multiple overlapping climatic hazards during the Maya Terminal Classic “megadroughts”. *Geophys. Res. Lett.* **41**, 5148–5157 (2014).

71. J. C. L. Chan, Frequency and intensity of landfalling tropical cyclones in East Asia: Past variations and future projections. *Meteorology* **2**, 171–190 (2023).

72. X. Chen, Y.-P. Guo, Z.-M. Tan, J. Zhao, Influence of different types of ENSO events on the tropical cyclone rainfall over the western North Pacific. *Climate Dynam.* **60**, 3969–3982 (2023).

73. W. Mei, S.-P. Xie, F. Primeau, J. C. McWilliams, C. Pasquero, Northwestern Pacific typhoon intensity controlled by changes in ocean temperatures. *Sci. Adv.* **1**, e1500014 (2015).

74. H. Xu, Y. Goldsmith, J. Lan, L. Tan, X. Wang, X. Zhou, J. Cheng, Y. Lang, C. Liu, Juxtaposition of western Pacific subtropical high on Asian summer monsoon shapes subtropical East Asian precipitation. *Geophys. Res. Lett.* **47**, e2019GL084705 (2020).

75. S. J. Camargo, H. Murakami, N. Bloemendaal, S. S. Chand, M. S. Deshpande, C. Dominguez-Sarmiento, J. J. González-Alemán, T. R. Knutson, I. I. Lin, I.-J. Moon, C. M. Patricola, K. A. Reed, M. J. Roberts, E. Scoccimarro, C. Y. Tam, E. J. Wallace, L. Wu, Y. Yamada, W. Zhang, H. Zhao, An update on the influence of natural climate variability and anthropogenic climate change on tropical cyclones. *Trop. Cyclone Res. Rev.* **12**, 216–239 (2023).

76. L. Wu, Z. P. Wen, R. H. Huang, R. G. Wu, Possible linkage between the monsoon trough variability and the tropical cyclone activity over the western North Pacific. *Mon. Weather Rev.* **140**, 140–150 (2012).

77. X. Fan, D. Zhao, Y. Li, X. Zhang, Y. Xie, L. Chen, Exploring synoptic patterns contributing to extreme rainfall from landfalling tropical cyclones in China. *Weather Clim. Extremes* **48**, 100768 (2025).

78. L. Chen, T. Wang, A. Sinha, F. Lin, H. Tang, H. Cheng, R. L. Edwards, L. Tan, A seasonally resolved stalagmite $\delta^{18}O$ record indicates the regional activity of tropical cyclones in Southeast China. *npj Clim. Atmos. Sci.* **7**, 168 (2024).

79. M. B. Osman, J. E. Tierney, J. Zhu, R. Tardif, G. J. Hakim, J. King, C. J. Poulsen, Globally resolved surface temperatures since the Last Glacial Maximum. *Nature* **599**, 239–244 (2021).

80. W. Mei, S.-P. Xie, Intensification of landfalling typhoons over the northwest Pacific since the late 1970s. *Nat. Geosci.* **9**, 753–757 (2016).

81. J. Schewe, S. N. Gosling, C. Reyer, F. Zhao, P. Ciais, J. Elliott, L. Francois, V. Huber, H. K. Lotze, S. I. Seneviratne, M. T. H. van Vliet, R. Vautard, Y. Wada, L. Breuer, M. Buechner, D. A. Carozza, J. Chang, M. Coll, D. Deryng, A. de Wit, T. D. Eddy, C. Folberth, K. Frieler, A. D. Friend, D. Gerten, L. Gudmundsson, N. Hanasaki, A. Ito, N. Khabarov, H. Kim, P. Lawrence, C. Morfopoulos, C. Mueller, H. M. Schmied, R. Orth, S. Ostberg, Y. Pokhrel, T. A. M. Pugh, G. Sakurai, Y. Satoh, E. Schmid, T. Stacke, J. Steenbeek, J. Steinkamp, Q. Tang, H. Tian, D. P. Tittensor, J. Volkholz, X. Wang, L. Warszawski, State-of-the-art global models underestimate impacts from climate extremes. *Nat. Commun.* **10**, 1005 (2019).

82. P. J. Reimer, W. E. N. Austin, E. Bard, A. Bayliss, P. G. Blackwell, C. Bronk Ramsey, M. Butzin, H. Cheng, R. L. Edwards, M. Friedrich, P. M. Grootes, T. P. Guilderson, I. Hajdas, T. J. Heaton, A. G. Hogg, K. A. Hughen, B. Kromer, S. W. Manning, R. Muscheler, J. G. Palmer, C. Pearson, J. van der Plicht, R. W. Reimer, D. A. Richards, E. M. Scott, J. R. Southon, C. S. M. Turney, L. Wacker, F. Adolphi, U. Büntgen, M. Capano, S. M. Fahrni, A. Fogtmann-Schulz, R. Friedrich, P. Köhler, S. Kudsk, F. Miyake, J. Olsen, F. Reinig, M. Sakamoto, A. Sookdeo, S. Talamo, The IntCal20 Northern Hemisphere radiocarbon age calibration curve (0–55 cal kBP). *Radiocarbon* **62**, 725–757 (2020).

83. C. Bronk Ramsey, Bayesian analysis of radiocarbon dates. *Radiocarbon* **51**, 337–360 (2009).

84. A. N. Williams, The use of summed radiocarbon probability distributions in archaeology: A review of methods. *J. Archaeol. Sci.* **39**, 578–589 (2012).

85. M. Ying, W. Zhang, H. Yu, X. Lu, J. Feng, Y. Fan, Y. Zhu, D. Chen, An overview of the China Meteorological Administration tropical cyclone database. *J. Atmos. Oceanic Tech.* **31**, 287–301 (2014).

86. J. Muñoz-Sabater, E. Dutra, A. Agustí-Panareda, C. Albergel, G. Arduini, G. Balsamo, S. Boussetta, M. Choulga, S. Harrigan, H. Hersbach, B. Martens, D. G. Miralles, M. Piles, N. J. Rodríguez-Fernández, E. Zsoter, C. Buontempo, J.-N. Thépaut, ERA5-Land: A state-of-the-art global reanalysis dataset for land applications. *Earth Syst. Sci. Data* **13**, 4349–4383 (2021).

87. J. He, K. Yang, W. Tang, H. Lu, J. Qin, Y. Chen, X. Li, The first high-resolution meteorological forcing dataset for land process studies over China. *Sci. Data* **7**, 25 (2020).

88. Q. Xiao, L. Chen, X. Zhang, Evaluations of BDA scheme using the advanced research WRF (ARW) model. *J. Appl. Meteorol. Climatol.* **48**, 680–689 (2009).

89. Q. Yan, T. Wei, R. L. Korty, J. P. Kossin, Z. Zhang, H. Wang, Enhanced intensity of global tropical cyclones during the mid-Pliocene warm period. *Proc. Natl. Acad. Sci. U.S.A.* **113**, 12963–12967 (2016).

90. J. Lu, S. Lou, X. Huang, L. Xue, K. Ding, T. Liu, Y. Ma, W. Wang, A. Ding, Stratospheric aerosol and ozone responses to the Hunga Tonga-Hunga Ha'apai volcanic eruption. *Geophys. Res. Lett.* **50**, e2022GL102315 (2023).

91. K. Emanuel, K. Oouchi, M. Satoh, H. Tomita, Y. Yamada, Comparison of explicitly simulated and downscaled tropical cyclone activity in a high-resolution global climate model. *J. Adv. Model. Earth Syst.* **2**, 9 (2010).

92. M. Guo, Ed. in *The Complete Collection of Oracle Bone Inscriptions* (Zhonghua Book Company, 1978–1983), vols. 1–13 (in Chinese).

93. Y. Li, "A study of anti-camality in Zhou dynasty," thesis, Jilin University, Changchun, China (2004) (in Chinese).

94. X. Lin, The origin of Sichuan wine: A study in the Shaman culture in ancient Sichuan. *South. Ethnol. Archaeol.* **1**, 73–85 (1987).

95. H. Sun, The burial of sacrificial pits in Sanxingdui: Burial nature, archaeology dating, pits owner and background. *South. Ethnol. Archaeol.* **17**, 9–13 (2013).

96. H. Sun, □□□□□□□□□□□. *Soc. Sci. China* **1**, 63–84 (2023) [The structure and characteristics of the Sanxingdui Polity].

97. H. Sun, Full-body bronze statues from Sanxingdui: Insights into clothing of the Sanxingdui people. *Jianghan Archaeology* **1**, 115–127 (2025).

98. X. Ren, J. Xu, H. Wang, M. Storozum, P. Lu, D. Mo, T. Li, J. Xiong, T. R. Kidder, Holocene fluctuations in vegetation and human population demonstrate social resilience in the prehistory of the Central Plains of China. *Environ. Res. Lett.* **16**, 055030 (2021).

99. T. Shi, Inundation of the Yellow River and the foreign military affairs of Bei-Song Dynasty. *Acad. J. Jinyang* **2**, 79–82 (2006).

100. Z. Dong, *Jiaguwen duandai yanjiu li* [Studies in the periodization of oracle bone script]. *Zhongyang Yanjiusuo Jikan Waibian* **1**, 323–424 (1933).

101. L. Zhou, Y. Yang, Z. Wang, J. Jia, L. Mao, Z. Li, X. Fang, S. Gao, Investigating ENSO and WPWP modulated typhoon variability in the South China Sea during the mid-late Holocene using sedimentological evidence from southeastern Hainan Island, China. *Mar. Geol.* **416**, 105987 (2019).

102. K.-F. Yu, J.-X. Zhao, Q. Shi, Q.-S. Meng, Reconstruction of storm/tsunami records over the last 4000 years using transported coral blocks and lagoon sediments in the southern South China Sea. *Quat. Int.* **195**, 128–137 (2009).

103. H. Dang, Z. Jian, F. Bassinot, P. Qiao, X. Cheng, Decoupled Holocene variability in surface and thermocline water temperatures of the Indo-Pacific Warm Pool. *Geophys. Res. Lett.* **39**, L01701 (2012).

104. D. Khider, C. S. Jackson, L. D. Stott, Assessing millennial-scale variability during the Holocene: A perspective from the western tropical Pacific. *Paleoceanography* **29**, 143–159 (2014).

105. L. Stott, K. Cannariato, R. Thunell, G. H. Haug, A. Koutavas, S. Lund, Decline of surface temperature and salinity in the western tropical Pacific Ocean in the Holocene epoch. *Nature* **431**, 56–59 (2004).

106. M. P. Erb, N. P. McKay, N. Steiger, S. Dee, C. Hancock, R. F. Ivanovic, L. J. Gregoire, P. Valdes, Reconstructing Holocene temperatures in time and space using paleoclimate data assimilation. *Clim. Past.* **18**, 2599–2629 (2022).

107. G. Yu, H. Shen, J. Liu, Impacts of climate change on historical locust outbreaks in China. *J. Geophys. Res.-Atmos.* **114**, D18104 (2009).

108. X. Yao, D. Xiao, *Collection and Classification of Inscriptions on Bones and Tortoise Shells in Yinxu* (Zhonghua Book Company, Beijing, 1989), pp. 694–696 (in Chinese).

109. W. Zhou, □□□□□□□□□□. *J. Chin.Hist. Geogr.* **1**, 185–196 (1999) [A research on the paleoclimate of the Yin times].

110. Y. Cai, X. Cheng, L. Ma, R. Mao, S. F. M. Breitenbach, H. Zhang, G. Xue, H. Cheng, R. L. Edwards, Z. An, Holocene variability of East Asian summer monsoon as viewed from the speleothem $\delta^{18}O$ records in central China. *Earth Planet. Sci. Lett.* **558**, 116758 (2021).

111. Y. Wang, H. Cheng, R. L. Edwards, Y. He, X. Kong, Z. An, J. Wu, M. J. Kelly, C. A. Dykoski, X. Li, The Holocene Asian monsoon: Links to solar changes and North Atlantic climate. *Science* **308**, 854–857 (2005).

112. C. Yin, W. Qiu, R. Li, Holocene paleofloods in the North China Plain. *J. Beijing Normal Univ.* **37**, 280–284 (2001).

113. H. Shen, L. Yu, H. Zhang, M. Zhao, Z. Lai, OSL and radiocarbon dating of flood deposits and its paleoclimatic and archaeological implications in the Yihe River Basin, East China. *Quat. Geochronol.* **30**, 398–404 (2015).

114. J. Shi, "Study on sedimentary environment of Holocene slackwater deposits in the Jiaozuo section of the Yellow River," thesis, Jiangsu Normal University, Xuzhou, China (2019) (in Chinese).

115. S.-Y. Yu, Z. Hou, X. Chen, Y. Wang, Y. Song, M. Gao, J. Pan, M. Sun, H. Fang, J. Han, T. R. Kidder, F.-H. Chen, Extreme flooding of the lower Yellow River near the Northgrippian-Meghalayan boundary: Evidence from the Shilipu archaeological site in southwestern Shandong Province, China. *Geomorphology* **350**, 106878 (2020).

116. X. Zhao, C. C. Huang, J. Pang, X. Zha, Y. Guo, G. Hu, Holocene climatic events recorded in palaeoflood slackwater deposits along the middle Yiluohe River valley, middle Yellow River basin, China. *J. Asian Earth Sci.* **123**, 85–94 (2016).

117. M. Yang, S. Wang, S. Kang, H. Liu, X. Wang, Optically stimulated luminescence dating of Sanyangzhuang profile, Henan Province. *J. Earth Environ.* **9**, 580–588 (2018).
